# Supporting self-care for eczema: protocol for two randomised controlled trials of ECO (Eczema Care Online) interventions for young people and parents/carers

**DOI:** 10.1136/bmjopen-2020-045583

**Published:** 2021-02-05

**Authors:** Ingrid Muller, Beth Stuart, Tracey Sach, Julie Hooper, Sylvia Wilczynska, Mary Steele, Kate Greenwell, Katy Sivyer, Lucy Yardley, Hywel C Williams, Joanne R Chalmers, Paul Leighton, Laura M Howells, Matthew J Ridd, Sandra Lawton, Gareth Griffiths, Jacqui Nuttall, Sinead M Langan, Amanda Roberts, Amina Ahmed, Hayden Kirk, Taeko Becque, Paul Little, Kim S Thomas, Miriam Santer

**Affiliations:** 1 School of Primary Care, Population Health and Medical Education, University of Southampton, Southampton, UK; 2 Norwich Medical School, University of East Anglia, Norwich, UK; 3 Department of Psychology, University of Southampton, Southampton, UK; 4 Department of Psychology, University of Portsmouth, Portsmouth, UK; 5 School of Experimental Psychology, University of Bristol, Bristol, UK; 6 Centre of Evidence Based Dermatology, University of Nottingham, Nottingham, UK; 7 Population Health Sciences, University of Bristol Faculty of Health Sciences, Bristol, UK; 8 Department of Dermatology, Rotherham NHS Foundation Trust, Rotherham, UK; 9 Southampton Clinical Trials Unit, University of Southampton, Southampton, UK; 10 Faculty of Epidemiology and Population Health, London School of Hygiene and Tropical Medicine, London, UK; 11 Neurological Rehabilitation, Solent NHS Trust, Southampton, UK

**Keywords:** eczema, primary care, world wide web technology

## Abstract

**Introduction:**

Eczema care requires management of triggers and various treatments. We developed two online behavioural interventions to support eczema care called ECO (Eczema Care Online) for young people and ECO for families. This protocol describes two randomised controlled trials (RCTs) aimed to evaluate clinical and cost-effectiveness of the two interventions.

**Methods and analysis:**

*Design*: Two independent, pragmatic, unmasked, parallel group RCTs with internal pilots and nested health economic and process evaluation studies. *Setting*: Participants will be recruited from general practitioner practices in England. *Participants*: Young people aged 13–25 years with eczema and parents and carers of children aged 0–12 years with eczema, excluding inactive or very mild eczema (five or less on Patient-Oriented Eczema Measure (POEM)). *Interventions*: Participants will be randomised to online intervention plus usual care or to usual eczema care alone. *Outcome measures*: Primary outcome is eczema severity over 24 weeks measured by POEM. Secondary outcomes include POEM 4-weekly for 52 weeks, quality of life, eczema control, itch intensity (young people only), patient enablement, health service and treatment use. Process measures include treatment adherence, barriers to adherence and intervention usage. Our sample sizes of 303 participants per trial are powered to detect a group difference of 2.5 (SD 6.5) in monthly POEM scores over 24 weeks (significance 0.05, power 0.9), allowing for 20% loss to follow-up. Cost-effectiveness analysis will be from a National Health Service and personal social service perspective. Qualitative and quantitative process evaluation will help understand the mechanisms of action and participant experiences and inform implementation.

**Ethics and dissemination:**

The study has been approved by South Central Oxford A Research Ethics Committee (19/SC/0351). Recruitment is ongoing, and follow-up will be completed by mid-2022. Findings will be disseminated to participants, the public, dermatology and primary care journals, and policy makers.

**Trial registration number:**

ISRCTN79282252.

**Strengths and limitations of this study**

- Two large randomised controlled trials of online complex behavioural interventions addressing an important clinical need and research gap to support eczema self-care.
- Comprehensive intervention development following the person-based approach with extensive input from young people and families with eczema.
- Both trials include qualitative and quantitative process evaluation to understand the interventions’ mechanisms of action and participant experiences.
- Cost-effectiveness of both interventions will be evaluated in nested health economic studies.
- Our primary outcome is self-reported eczema severity using the Patient-Oriented Eczema Measure (POEM), but the lack of assessment of objective eczema severity could be viewed as a limitation.

## Introduction

### Background and rationale

Eczema can cause substantial impact on quality of life, primarily because of sleep disturbance and itch.^1^ Families of children with eczema express frustration that they do not receive enough information about how to manage the condition,^2^ as do adults with eczema.^3^ The National Institute for Health and Care Excellence (NICE) guidance on eczema^4^ highlights that the main cause of treatment failure is non-adherence and there is a need for new ways to support adherence.^5^ Reasons for non-adherence include therapy being time-intensive,^6 7^ lack of understanding of treatments and how to use them,^6^ underuse of topical corticosteroids related to concerns about side effects,^8^ conflicting advice from different health professionals regarding how to use topical corticosteroids^9 10^ and child refusal.^7^

Self-care includes all the health behaviours needed to look after one’s own condition. Non-adherence is related to people’s understanding of their condition and its treatment, as well as perceived need for treatments and concerns about adverse consequences of treatments.^11^ Self-care is particularly complex in eczema as it involves regular application of topical treatments (mainly emollients for maintenance and topical corticosteroids for inflamed eczema) and avoidance of triggers (eg, soap). At present, many people and families receive little advice on how to manage the condition, or obtain advice of variable quality from the internet.^12^ There is a need for high-quality, accessible interventions, as well as evidence of whether interventions work so that, if effective, clinicians can signpost towards these as an essential part of routine care.

Currently, 96% of British households have access to the internet, with 99% of adults being regular internet users.^13^ Although information about eczema is widely available on the internet, it is of variable quality, often promoting commercial products of unproven efficacy. Patients and parents/carers find it difficult to know which information is reliable.^12^

We have developed two web-based interventions to support eczema management: ECO (Eczema Care Online) for parents and carers of children aged 0–12 years with eczema, and ECO for young people aged 13–25 years with eczema. Parents of children with eczema and young people with eczema are likely to have different support and information needs. We have therefore developed two separate interventions to be evaluated in two separate randomised controlled trial (RCTs). This paper provides an abridged version of the full protocol that is available on the project website.^14^

### Study objectives

The primary objective is to determine the clinical effectiveness of two online interventions compared with usual care for eczema: one for young people aged 13–25 with eczema (ECO-YP) and one for parents and carers of children aged 0–12 with eczema (ECO-PC).

The secondary objectives are (1) to determine the cost-effectiveness of the online interventions from a National Health Service (NHS) and personal social service perspective and (2) to determine the interventions’ mechanisms of action and factors related to participant engagement and treatment adherence and its outcomes.

### Trial design

This protocol comprises two independent pragmatic, parallel group 1:1 allocation individually randomised superiority trials:

1. ECO-YP: to assess the effectiveness of an online intervention in young people (YP) with eczema aged 13–25 years as measured by Patient-Oriented Eczema Measure (POEM) 4-weekly scores over 24 weeks.
2. ECO-PC: to assess the effectiveness of an online intervention in parents and carers (PC) of children with eczema aged 0–12 years as measured by POEM 4-weekly scores over 24 weeks.

Total duration of follow-up will be 52 weeks with primary outcome assessed over the first 24 weeks.

## Methods and analysis

### Study setting

Primary care (general practitioner (GP) surgeries) in Wessex, West of England, East Midlands, and Thames Valley and South Midlands.

### Recruitment

We will identify children with eczema aged 0–12 years and young people with eczema aged 13–25 years via an electronic records search developed by the study team and run by staff at the participating GP surgeries. A doctor or delegated member of the practice team will screen the identified list to assess suitability to receive a study invitation. Potential participants will be sent an invitation pack containing the study URL and a unique code to register if they would like to take part. After registering on the intervention website, the participants will be asked to provide informed consent and complete screening and baseline measures.

Parents or legal representatives of potential participants for ECO-YP aged 13–15 years will be sent information about the study and a URL to provide online consent if they are happy for their child to take part. On receipt of parental consent, children aged 13–15 years old will be sent a participant invitation pack with the intervention website URL and unique ID to sign up if they would like to take part. Once registered, they will be asked to assent online.

### Eligibility criteria

Eligibility for inclusion in ECO-YP: aged 13–25 years; identified from GP records as having eczema and have obtained a prescription for eczema treatment (emollient or topical corticosteroid) in the past 12 months; POEM score greater than 5, to include mild to severe eczema, but exclude those with very mild or inactive eczema to avoid floor effects; have internet access.

Eligibility for inclusion in ECO-PC: parent/carer of a child aged 0–12 years; child identified from GP records as having eczema and has obtained a relevant prescription in the past 12 months; child has a POEM score greater than 5, to include mild to severe eczema, but exclude those with very mild or inactive eczema; have internet access.

Only one person per household can take part in the trials. If a parent or carer has more than one child who meets the inclusion criteria, they will be asked to specify one child to participate.

**Figure 1:**
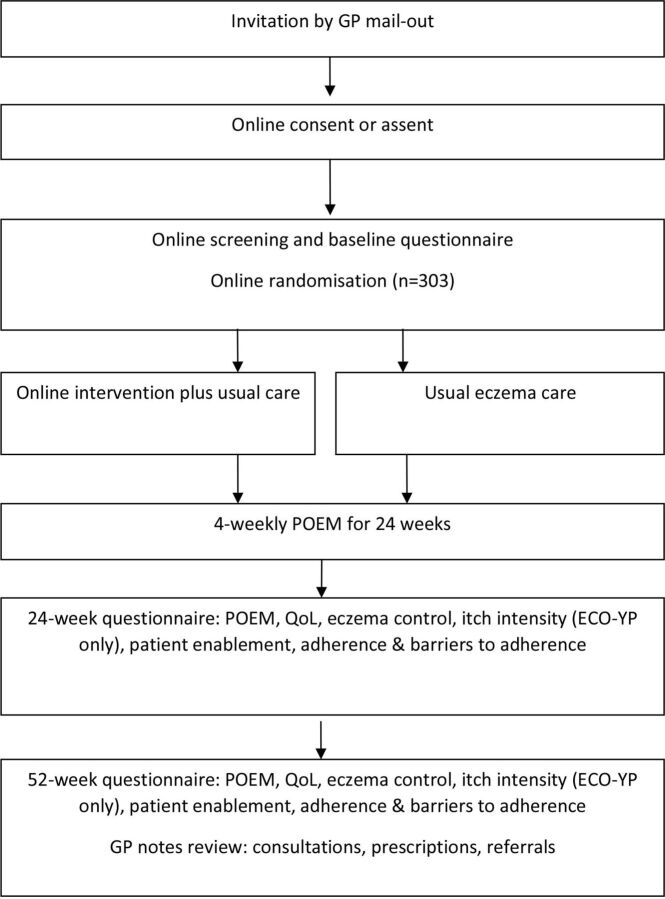
Flow of participants through the trial. GP, general practitioner; POEM, Patient-Oriented Eczema Measure; QoL, quality of life.

Potential participants from ECO-YP and ECO-PC are excluded if: unable to give informed consent; unable to read and write English, as the intervention content and outcome measures are in English; have taken part in another eczema study in the past 3 months; took part in think aloud interviews as part of ECO intervention development.^15^ Qualitative interviewees who did not view intervention materials will not be excluded. See figure 1 for participant timeline.

### Randomisation procedures and blinding

Participants will complete informed consent or assent and baseline questionnaires online within the intervention developed using LifeGuide software.^16^ Those who do not meet the eligibility criteria of a minimum POEM score greater than 5 are presented with information explaining that they are not eligible for the study and signposted to other resources.

Eligible participants are randomised online to either (1) usual eczema care or (2) online intervention plus usual care through LifeGuide software. Randomisation is carried out in blocks and stratified by age (13–17; 18–25 (ECO-YP), and 0–5; 6–12 (ECO-PC)), baseline eczema severity (POEM scores 6–7 (mild); 8–16 (moderate); 17–28 (severe)) and recruitment region as these may influence how participants engage with the interventions.

It is not possible to mask participants to their allocation group. Participants are informed online as to which group they have been allocated to immediately after randomisation and are notified by email. The immediate trial team dealing with participant queries will have access to group allocation, but the wider Trial Management Group and trial statistician will remain blinded.

### Intervention and group details

#### Usual care group

Participants randomised to usual care will continue to receive their usual medical advice and prescriptions. They can seek online support but will not be supported in doing so by the study team and will not have access to the online interventions during their participation in the trial. Participants allocated to the usual care group will be given access to the intervention after 52-week follow-up is complete.

#### Behavioural intervention groups (ECO-YP and ECO-PC)

Participants randomised to the intervention group will receive access to an online behavioural intervention to support eczema self-care in addition to usual eczema care, as above. The interventions were developed following the person-based approach to intervention development^17 18^ to ensure they are meaningful, optimally engaging and relevant to target users, and draws on a theoretical framework including the Extended-Common Sense Model,^19^ Social Cognitive Theory,^20^ the Behaviour Change Wheel and associated Theoretical Domains Framework.^21^ All intervention content is evidence-based, and the interventions are tailored and include interactive and audio-visual features. The interventions were initially developed by the research team consisting of behavioural psychologists, patient representatives, clinicians (GPs, dermatology nurse consultant, dermatologists) and skin researchers before being optimised through extensive user feedback to ensure they are acceptable, feasible and optimally engaging to target users.^22^

The online interventions target core behaviours linked to eczema management:

- Regular use of emollients and appropriate use of topical corticosteroids.
- Avoiding eczema irritants and triggers.
- Minimising scratching.
- Emotional management.

The interventions use behavioural techniques to promote adherence and support eczema self-care by building on aspects like knowledge, skills, self-efficacy, social support and environmental factors such as social and physical opportunity.

The interventions take participants through a core section before giving access to the main menu with the choice of various topics of interest to young people and families with eczema. These topics include eczema treatments, infections, talking to your healthcare professional, diet and allergy, sleep and itch, physical activity, coping with stress and transitioning to self-care. The interventions also include a ‘2-week challenge’ where participants are encouraged to use their eczema treatment regularly for 2 weeks, supported by optional text and email reminders and support. Intervention content has been developed to be interactive and engaging, with tailoring to suggest topics that may be of relevance. The intervention also contains a series of animated videos focussing on the core target behaviours.

ECO-YP has been developed for people aged 13–25 years with eczema. The intervention covers the topics mentioned above, as well as additional topics that are important particularly to this age group, such as information about finances, school, university, or work and cosmetics.

ECO-PC has been developed for parents of children aged 0–12 years with eczema. This intervention covers the same wide range of topics relevant to eczema, as well as sections that are specifically relevant to parents and co-management of eczema, such as transitioning to co-management, dealing with child resistance and managing your child’s eczema at nursery and school. Intervention description follows TIDieR guidelines^23^; detailed intervention development and optimisation studies will be published separately.

### Outcomes

All participant-reported outcome measures and intervention usage data are collected online via LifeGuide software. Outcome measures are similar across ECO-YP and ECO-PC, where there are differences, these are highlighted (table 1). POEM, RECAP and itch intensity measures have been recommended as core outcome measures for eczema by the international Harmonising Outcome Measures for Eczema group.^24 25^

**Table 1:** Schedule of observations

Outcomes collected	Baseline	24 weeks (primary outcome)	52 weeks (end of study)
Baseline characteristics			
Demographics	✓		
Prior belief about effectiveness	✓		
Previous online resource use	✓		
Clinical effectiveness outcomes			
POEM (4-weekly)	✓	✓	✓
Long-term control (Recap)	✓	✓	✓
Itch intensity measure (ECO-YP only)	✓	✓	✓
Patient Enablement Instrument (PEI)	✓	✓	✓
Cost-effectiveness outcomes			
CHU-9D (ECO-PC for parents/carers of children aged 2–12 only)	✓	✓	✓
EQ-5D-5L (ECO-YP only)	✓	✓	✓
Medical notes review for medication use, service use and referrals		✓ (including 3 months pre-baseline period)	
Process outcomes			
Problematic Experiences of Therapy Scale (PETS)	✓	✓	✓
Frequency of eczema treatment use (adherence)	✓	✓	✓
Intervention usage		✓ (recorded throughout study)	

POEM, Patient-Oriented Eczema Measure.

#### Primary outcome

The primary outcome for both trials is the difference in patient-reported eczema severity between the intervention and usual care group as measured by POEM, every 4 weeks over 24 weeks.^26 27^

POEM includes seven questions about the frequency of eczema symptoms over the previous week that are summed to give a score from 0 (no eczema) to 28 (worst possible eczema). POEM can be completed by young people and children or by proxy (carer report, ECO-PC), demonstrates good validity, test–retest reliability and responsiveness to change.^28^

#### Secondary outcomes

Secondary outcomes include (1) difference in POEM scores 4-weekly over 52 weeks; (2) quality of life at 24 and 52 weeks, measured in ECO-YP, using the EQ-5D-5L^29^ self-completed by the young person, and in ECO-PC by proxy using the Child Health Utility-Nine Dimensions (CHU-9D)^30^ for children aged 2–12 years; (3) eczema control at 24 and 52 weeks, measured by RECAP (Recap for atopic eczema patients)^31^; (4) itch intensity^32^ at 24 and 52 weeks, measured as worst itch in the last 24 hours (not validated for proxy completion for children, and therefore used in ECO-YP only); (5) patient enablement at 24 and 52 weeks, the self-perceived ability to understand and cope with health issues, will be measured using the Patient Enablement Instrument (PEI)^33^; (6) health service use and medication use, measured by medical notes review for the 3-month period prior to baseline and the whole 52-week trial period; (7) cost-effectiveness combining quality of life and health service use and medication use.

#### Other measures

Prior belief about the effectiveness of the intervention and online resource use (websites or apps) for eczema will be measured at baseline and will be used in a planned subgroup analysis to explore whether there is an interaction between prior belief, online resource use and treatment effectiveness.

#### Process measures

Self-reported barriers to adherence to eczema treatments will be measured at 24 and 52 weeks using the Problematic Experiences of Therapy Scale (PETS)^34^ and frequency of eczema treatment use (treatment adherence) will be measured by self-report. Intervention usage data for each participant will be automatically recorded by LifeGuide software for the duration of the 52-week trial period.

### Internal pilot phase

The first 3 months of participant recruitment was an internal pilot phase to test trial procedures, which mirrored the main trial protocol exactly. We assessed study uptake, recruitment and follow-up procedures, randomisation and participant engagement in accessing the intervention. Success criteria for the pilot phase are listed in the full protocol (available from ECO website).^14^

### Data collection methods and retention

All study procedures are automated and carried out online through the LifeGuide software.^16^ Participants wishing to take part in the study provide consent and assent (where required) and complete an online baseline questionnaire before being randomised to either the usual care group or the intervention group. Participants in the intervention group then have access to the intervention website (either ECO-YP or ECO-PC).

All participants are asked to complete a 4-weekly POEM questionnaire online for 52 weeks. Participants are also asked to complete a longer 24-week and 52-week follow-up questionnaire online. When signing up for the trial participants are asked if they would prefer reminders by email, text message or both. Automated emails and/or text messages are sent to notify participants when their follow-up questionnaires are available for completion. Reminders will be sent to non-responders after 5 days (and after 10 days for 24-week and 52-week questionnaires), followed by reminder telephone calls approximately 4 days later from the research team, at which point participants will be invited to complete selected follow-up questions over the phone.

### Sample size

The sample size calculation for ECO-YP and ECO-PC is based on 4-weekly POEM scores using repeated measures over the first 24 weeks of the trial, seeking to detect a minimum clinically important difference (MCID) of 2.5 points between groups (SD 6.5). Assuming a correlation between repeated measures of 0.70, with 90% power and 5% significance, this requires a total sample size of 121 per group in each of the two trials. Allowing for 20% loss to follow-up gives a total sample size of 303 in each of the two trials. The sample size was amended during the trial, see the Protocol amendments section for details.

### Statistical analysis plan

Primary analyses of the ECO-YP and ECO-PC trials will be generalised linear mixed models, allowing for observations nested within participants over time. All analyses will control for key covariates, including age and baseline eczema severity, and will be set out in full in the statistical analysis plan prior to database lock. For secondary outcome measures, linear models will be used for continuous outcomes. Where the assumptions for linear models are not met, we will use other appropriate distributions or non-parametric methods if no suitable distribution can be found. Logistic regression will be used for binary outcome measures.

We will collect data on use of other websites at the start and end of the trials to check whether there is a difference between groups in accessing other eczema sites and plan sensitivity analyses to examine whether accessing other resources affects outcomes. All trials of online interventions must assume that users in both groups may access other websites, and so trials provide a useful test of whether the intervention being evaluated is superior to the websites users can already access.

All analyses will be on an intention-to-treat basis (analysed as randomised), detailed in the statistical analysis plan, and include participants from the internal pilots and full RCTs. No interim analyses are planned. The structure and pattern of missing data will be examined, if appropriate, and a sensitivity analysis based on data imputed using a multiple imputation model presented. Findings will be reported in accordance with the CONSORT (Consolidated Standards of Reporting Trials) statement.

### Health economic evaluation

Two within trial economic evaluations will estimate whether ECO-YP and ECO-PC are cost-effective compared with usual care from an NHS and personal social services perspective. We will estimate the cost of the interventions and collect data on wider resource use (primary care, secondary care and accident and emergency use) and eczema-related prescriptions through medical notes review. Resource items will be valued using published unit costs for the most recent common price year to the time of analysis.

There is currently no agreed approach to valuing health outcomes in children in economic evaluations and there has been limited use of child and adolescent population-specific measures to generate health state utilities in NICE technology assessments.^35^ In ECO-PC for parents and carers of children aged 0–12, we will collect by proxy the CHU-9D,^36^ a paediatric generic preference-based instrument, in those aged 2 and older. Although the CHU-9D was developed for children aged 7 and older, its completion by proxy in younger age groups is currently being trialled^36^ and the developer of the instrument has given us additional guidance to use with parents and carers with children in this age group. This approach is being taken as only parents and carers are expected to interact with the intervention.

In the ECO-YP (young people aged 13–25), all participants are asked to self-complete the EQ-5D-5L in order to estimate their health-related quality of life. To prevent any discontinuity, the EQ-5D-Y will not be used in those under the age of 16 as this is a different instrument from the EQ-5D-5L.^37 38^ All participants will be asked to complete the EQ-5D-5L at baseline, 24 and 52 weeks and the scores from these will be converted to utility scores using UK preference weights in line with current recommendations at the time of the analysis.^37 39^ Following this, the utility values will be used to estimate quality-adjusted life years (QALY) for the trial period using linear interpolation and area under the curve with and without baseline adjustment.^40^

Cost-effectiveness (using change in POEM between baseline and 52 weeks, secondary analysis) and cost utility analyses (primary analysis) will be performed. Costs and benefits will not be discounted given the 12-month timeframe. Using information on costs and benefits, regression analysis will be conducted to estimate the incremental cost, incremental benefit and incremental cost utility of the online intervention compared with usual care (over the trial period). If one arm is clearly dominant (less costly and more effective), a recommendation can be made on this basis. If non-dominance occurs (that is if costs are greater and the intervention is more effective or if the intervention is cheaper and less effective), an incremental cost-effectiveness ratio will be produced and a judgement about value for money will need to be made. The economic evaluation will be undertaken and analysed in line with guidelines.^41 42^ Missing data will be dealt with in line with the approach taken in the main clinical statistical analysis, with sensitivity analysis undertaken to test the impact of approach if missing data is a particular problem. A detailed health economic analysis plan will be written and reviewed before the trial database is locked.

### Nested process evaluation

The nested process evaluation studies are being carried out to understand intervention processes and participants’ experiences of using the interventions.

#### Quantitative process evaluation

We will use baseline data to examine potential predictors and moderator effects of participant characteristics (eg, age, eczema symptom severity, baseline attitudes) on intervention engagement (objectively recorded detailed website usage and self-reported treatment adherence) and outcome. We will also assess and analyse hypothesised mediators of treatment adherence and intervention outcomes; specifically changes in beliefs about treatment (PETS) as well as intervention usage. Objective measures of intervention usage are automatically recorded (with informed participant consent), allowing evaluations of usage patterns, such as time spent on intervention, number of visits to the intervention website and pages visited.

#### Qualitative process evaluation

Qualitative process interviews will be carried out with approximately 30–40 participants (15–20 from ECO-YP and 15–20 from ECO-PC, or until saturation of the main themes are achieved). These interviews will provide in-depth understanding of patient and carers’ experiences within the trial and provide a better understanding of factors that may influence engagement.

Interviews will be conducted via telephone or video call by a member of the research team experienced in qualitative research methods. We will interview participants from the intervention group and the usual care group and use purposive sampling to ensure a range of age, gender, ethnicity, eczema severity, website usage, deprivation index and region. Potential participants will be contacted after being in the trial for at least 3 months by a member of the research team to check whether they would like to take part in an interview or have any questions about the study. Participants will be asked to give their consent online prior to the interview. Interviews will use a combination of open-ended and focused questions and be transcribed verbatim.

Qualitative data will first be analysed using inductive thematic analysis.^43^ We will then explore how emerging themes may map onto theoretical frameworks in order to relate our insights to generalisable theoretical constructs and inform implementation planning.

#### Process evaluation analysis

We will triangulate findings from the quantitative and qualitative process analyses^44^ to explore and test the causal mechanisms proposed, to help inform interpretation of trial results, and determine how the interventions could be improved and how implementation into clinical practice could be facilitated.

### Patient and public involvement

The study team includes two patient and public involvement (PPI) members (AR and AA) who have been involved in the project from the earliest stages. They are involved in all aspect of the ECO programme and trials, including intervention development,^22^ trial design, attending trial and programme management meetings, protocol discussions, developing participant facing materials and coauthoring outputs. Our PPI partners will also be key for dissemination and future implementation.

## Ethics and dissemination

The study has received the favourable opinion of South Central–Oxford A Research Ethics Committee (19/SC/0351). This summary protocol is based on approved protocol v3 (20/05/2020), ISRCTN reference 79 282 252.

### Data monitoring

An independent Programme Steering Committee take responsibility for safeguarding the interest of study participants, monitor the main outcome measures including safety and efficacy, and monitor the overall conduct of the trials.

### Protocol amendments

One substantial protocol amendment has been made since initial ethics approval (Substantial Amendment 1, number 47369.A4, approved 01/06/20). This amendment was in response to the COVID-19 pandemic in order to:

1. Make all trial process online. The original protocol required parents or guardians of 13–15 year olds to return parental consent by post.
2. Increase in sample size. Our original sample size was 200 participants per trial, based on the published POEM MCID of 3.^45^ However, research has since suggested that a smaller POEM MCID may be meaningful in certain contexts.^46^ Recruitment to both trials exceeded expectation and a protocol amendment was made to change the sample size to a minimum of ‘200 participants’ per trial to allow us to continue recruitment while a revised sample size was discussed with our Trial Management Group, Programme Management Group, Programme Steering Committee and funder, without access to study outcome data or any interim analysis. The final agreed sample size for the trials were based on seeking to detect a POEM MCID of 2.5 points between groups, based on two repeated measures (SD 6.5), allowing for 20% loss to follow-up requiring sample size of 303 participants in each of the two trials.

### Dissemination

As a minimum, study progress, outputs and trial findings will be made available via the study website^14^ and project twitter (@ECO_eczema). Summaries will also be sent to participants and participating GP surgeries. Findings will be presented at conferences and published in peer-reviewed journals. We will make available a deidentified dataset on request.

## Supplementary Material

Reviewer comments

Author's manuscript
