# Supplementary material for: Supporting self-care for eczema: protocol for two randomised controlled trials of ECO (Eczema Care Online) interventions for young people and parents/carers
Source: BMJ Open. 2021 Feb 5;11(2):e045583. doi: 10.1136/bmjopen-2020-045583 (PMC7925854; doi:10.1136/bmjopen-2020-045583)
Supplement: Author's manuscript [file bmjopen-2020-045583.draft_revisions.pdf]

BMJ Open is committed to open peer review. As part of this commitment we make the peer review history of every article we publish publicly available.

When an article is published we post the peer reviewers' comments and the authors' responses online. We also post the versions of the paper that were used during peer review. These are the versions that the peer review comments apply to.

The versions of the paper that follow are the versions that were submitted during the peer review process. They are not the versions of record or the final published versions. They should not be cited or distributed as the published version of this manuscript.

BMJ Open is an open access journal and the full, final, typeset and author-corrected version of record of the manuscript is available on our site with no access controls, subscription charges or pay-per-view fees (http://bmjopen.bmj.com).

If you have any questions on BMJ Open's open peer review process please email info.bmjopen@bmj.com

BMJ Open

Supporting self-care for eczema: protocol for two randomised controlled trials of ECO (Eczema Care Online) interventions for young people and parents/carers

Journal:	*BMJ Open*
Manuscript ID	bmjopen-2020-045583
Article Type:	Protocol
Date Submitted by the Author:	05-Oct-2020

Complete List of Authors:	Muller, Ingrid; University of Southampton, School of Primary Care, Population Health and Medical Education<br>Stuart, Beth; University of Southampton, School of Primary Care, Population Health and Medical Education<br>Sach, Tracey; University of East Anglia, Norwich Medical School<br>Hooper, Julie; University of Southampton, School of Primary Care, Population Health and Medical Education<br>Wilczynska, Sylvia; University of Southampton, School of Primary Care, Population Health and Medical Education,<br>Steele, Mary; University of Southampton School of Psychology,<br>Greenwell, Kate; University of Southampton, Department of Psychology<br>Sivyer, Katy; University of Southampton, Department of Psychology; University of Portsmouth, Department of Psychology<br>Yardley, Lucy; University of Southampton, Department of Psychology; University of Bristol, School of Experimental Psychology<br>Williams, HC; University of Nottingham, Centre of Evidence Based Dermatology<br>Chalmers, Joanne; University of Nottingham, Centre of Evidence Based Dermatology<br>Leighton, Paul; University of Nottingham, Centre of Evidence Based Dermatology<br>Howells, Laura; University of Nottingham Centre for Evidence Based Dermatology,<br>Ridd, Matthew; University of Bristol Faculty of Health Sciences, Population Health Sciences;<br>Lawton, Sandra; Rotherham NHS Foundation Trust, Dermatology Department<br>Griffiths, Gareth; University of Southampton, Southampton Clinical Trials Unit<br>Nuttall, Jacqui; University of Southampton, Southampton Clinical Trials Unit<br>Langan, Sinead; London School of Hygiene & Tropical Medicine,<br>Roberts, Amanda; University of Nottingham, Centre of Evidence-Based Dermatology<br>Ahmed, Amina; University of Nottingham, Centre of Evidence Based Dermatology<br>Kirk, Hayden; Solent NHS Trust, Neurological Rehabilitation<br>Little, Paul; University of Southampton, School of Primary Care, Population Health and Medical Education

Thomas, Kim; University of Nottingham, Centre of Evidence Based Dermatology
Santer, Miriam; University of Southampton, School of Primary Care, Population Health and Medical Education

Keywords: Eczema < DERMATOLOGY, PRIMARY CARE, World Wide Web technology < BIOTECHNOLOGY & BIOINFORMATICS

SCHOLARONE™ Manuscripts

I, the Submitting Author has the right to grant and does grant on behalf of all authors of the Work (as defined in the below author licence), an exclusive licence and/or a non-exclusive licence for contributions from authors who are: i) UK Crown employees; ii) where BMJ has agreed a CC-BY licence shall apply, and/or iii) in accordance with the terms applicable for US Federal Government officers or employees acting as part of their official duties; on a worldwide, perpetual, irrevocable, royalty-free basis to BMJ Publishing Group Ltd ("BMJ") its licensees and where the relevant Journal is co-owned by BMJ to the co-owners of the Journal, to publish the Work in this journal and any other BMJ products and to exploit all rights, as set out in our licence.

The Submitting Author accepts and understands that any supply made under these terms is made by BMJ to the Submitting Author unless you are acting as an employee on behalf of your employer or a postgraduate student of an affiliated institution which is paying any applicable article publishing charge ("APC") for Open Access articles. Where the Submitting Author wishes to make the Work available on an Open Access basis (and intends to pay the relevant APC), the terms of reuse of such Open Access shall be governed by a Creative Commons licence – details of these licences and which Creative Commons licence will apply to this Work are set out in our licence referred to above.

Other than as permitted in any relevant BMJ Author's Self Archiving Policies, I confirm this Work has not been accepted for publication elsewhere, is not being considered for publication elsewhere and does not duplicate material already published. I confirm all authors consent to publication of this Work and authorise the granting of this licence.

For peer review only

Supporting self-care for eczema: protocol for two randomised controlled trials of ECO (Eczema Care Online) interventions for young people and parents/carers

Ingrid Muller[1], Beth Stuart[1], Tracey H Sach[2], Julie Hooper[1], Sylvia Wilczynska[1], Mary Steele[1], Kate Greenwell[3], Katy Sivyer[34], Lucy Yardley[3, 5], Hywel C Williams[6], Joanne R Chalmers[6], Paul Leighton[6], Laura Howells[6], Matthew J Ridd[7], Sandra Lawton[8], Gareth Griffiths[9], Jacqui Nuttall[9], Sinéad M Langan[10], Amanda Roberts[11], Amina Ahmed[6], Hayden Kirk[12], Paul little[1], Kim S Thomas[6], Miriam Santer[1]

[1] School of Primary Care, Population Health and Medical Education, University of Southampton, UK

[2] Norwich Medical School, University of East Anglia, Norfolk, UK

[3] Centre for Clinical and Community Applications of Health Psychology, University of Southampton, Southampton, UK

[4] Department of Psychology, Faculty of Science and Health, University of Portsmouth, UK

[5] School of Experimental Psychology, University of Bristol, Bristol, UK

[6] Centre of Evidence Based Dermatology, School of Medicine, University of Nottingham, Nottingham, UK

[7] Population Health Sciences, University of Bristol, Bristol, UK

[8] Rotherham NHS Foundation Trust, Rotherham, UK

[9] Southampton Clinical Trials Unit, University of Southampton, Southampton, UK

[10] Faculty of Epidemiology and Population Health, London School of Hygiene and Tropical Medicine, London, UK

[11] Nottingham Support Group for Carers of Children with Eczema, Nottingham, UK

[12] Solent NHS Trust, Southampton, UK

Word count: 3992

Keywords: Atopic eczema / dermatitis, behavioural intervention, RCT, online, self-management, cost-effectiveness

Corresponding author:

Ingrid Muller

School of Primary Care, Population Health and Medical Education, University of Southampton, UK

I.Muller@soton.ac.uk

For peer review only - http://bmjopen.bmj.com/site/about/guidelines.xhtml

Abstract

Introduction

Eczema care requires management of triggers and various treatments. We developed two online behavioural interventions to support eczema care called ECO (Eczema Care Online) for young people and ECO for families. This protocol describes two randomised controlled trials aimed to evaluate clinical and cost effectiveness of the two interventions.

Methods and analysis

Design: Two independent, pragmatic, unmasked, parallel group randomised controlled trials with internal pilots and nested health economic and process evaluation studies. *Setting*: Participants will be recruited from GP practices in England. *Participants*: young people aged 13-25 years with eczema and parents / carers of children aged 0-12 years with eczema, excluding inactive or very mild eczema (5 or less on Patient-Oriented Eczema Measure (POEM)). *Interventions*: Participants will be randomised to online intervention plus usual care or to usual eczema care alone. *Outcome measures*: Primary outcome is eczema severity over 24 weeks measured by POEM. Secondary outcomes include: POEM 4-weekly for 52 weeks, quality of life, eczema control, itch intensity (young people only), patient enablement, health service and treatment use. Process measures include treatment adherence, barriers to adherence, and intervention usage. Our sample sizes of 303 participants per trial are powered to detect a group difference of 2.5 (SD 6.5) in monthly POEM scores over 24 weeks (significance 0.05, power 0.9), allowing for 20% loss to follow-up. Cost effectiveness analysis will be from an NHS and personal social service perspective. Qualitative and quantitative process evaluation will help understand mechanisms of action and participant experiences and inform implementation.

Ethics and dissemination

The study has been approved by South Central Oxford A Research Ethics Committee (19/SC/0351). Recruitment is ongoing, and follow-up will be completed by mid-2022. Findings will be disseminated to participants, the public, dermatology and primary care journals, and policymakers.

Trial registration number: ISRCTN79282252

ARTICLE SUMMARY

Strengths and limitations of this study

- Two large RCTs of online complex behavioural interventions addressing an important clinical need and research gap to support eczema self-care.
- Comprehensive intervention development following the Person-Based Approach with extensive input from young people and families with eczema.
- Both trials include an internal pilot phase to evaluate feasibility and patient reported outcomes.
- Cost-effectiveness of both interventions will be evaluated in nested health economic studies.
- Detailed intervention usage data will be collected via LifeGuide software and both trials will undergo a qualitative and quantitative process evaluation to understand the interventions' mechanisms of action and participant experiences.

For peer review only

INTRODUCTION

Background and rationale

Eczema can cause substantial impact on quality of life, primarily because of sleep disturbance and itch.[1] Families of children with eczema express frustration that they do not receive enough information about how to manage the condition,[2] as do adults with eczema.[3] NICE guidance on eczema [4] highlights that the main cause of treatment failure is non-adherence and there is a need for new ways to support adherence.[5] Reasons for non-adherence include therapy being time-intensive [6,7], lack of understanding of treatments and how to use them [6], under-use of topical corticosteroids related to concerns about side-effects [8], conflicting advice from different health professionals regarding how to use topical corticosteroids[9,10], and child-refusal[7].

Self-care includes all the health behaviours needed to look after one's own condition. Non-adherence is related to people's understanding of their condition and its treatment, as well as perceived need for treatments and concerns about adverse consequences of treatments.[11] Self-care is particularly complex in eczema as it involves regular application of topical treatments (mainly emollients for maintenance and topical corticosteroids for inflamed eczema) and avoidance of triggers (e.g. soap). Presently, many people / families receive little advice on how to manage the condition, or obtain advice of variable quality from the internet.[12] There is a need for high quality, accessible interventions, as well as evidence of whether interventions work so that, if effective, clinicians can signpost towards these as an essential part of routine care.

Currently, 96% of British households have access to the internet with 99% of adults being regular internet users.[13] Although information about eczema is widely available on the internet, it is of variable quality, often promoting commercial products of unproven efficacy. Patients and parents/carers find it difficult to know which information is reliable.[12]

We have developed two web-based interventions to support eczema management; ECO (Eczema Care Online) for parents and carers of children aged 0-12 years with eczema, and ECO for young people aged 13-25 years with eczema. Parents of children with eczema and young people with eczema are likely to have different support and information needs. We have therefore developed two separate interventions to be evaluated in two separate RCTs. This paper provides an abridged version of the full protocol that is available on the project website[14].

Study objectives

The primary objective is to determine the clinical effectiveness of two online interventions compared to usual care for eczema: one for young people aged 13-25 with eczema (ECO-YP) and one for parents/carers of children aged 0-12 with eczema (ECO-PC).

Secondary objectives are: i) to determine the cost effectiveness of the online interventions from a National Health Service (NHS) and personal social service perspective, and ii) to determine the interventions' mechanisms of action and factors related to participant engagement and treatment adherence and its outcomes.

Trial design

This protocol comprises two independent pragmatic, parallel group 1:1 allocation individually randomised superiority trials:

For peer review only - http://bmjopen.bmj.com/site/about/guidelines.xhtml

1. ECO-YP: to assess the effectiveness of an online intervention in young people (YP) with eczema aged 13-25 years as measured by Patient-Oriented Eczema Measure (POEM) 4-weekly scores over 24 weeks.
2. ECO-PC: to assess the effectiveness of an online intervention in parents and carers (PC) of children with eczema aged 0-12 years as measured by Patient-Oriented Eczema Measure (POEM) 4-weekly scores over 24 weeks.

Total duration of follow-up will be 52 weeks with primary outcome assessed over the first 24 weeks.

METHODS AND ANALYSIS

Study setting

Primary care (General practitioner (GP) surgeries) in Wessex, West of England, East Midlands, and Thames Valley and South Midlands.

Recruitment

We will identify children with eczema aged 0-12 years and young people with eczema aged 13-25 years via an electronic records search developed by the study team and run by staff at the participating GP surgeries. A doctor or delegated member of the practice team will screen the identified list to assess suitability to receive a study invitation. Potential participants will be sent an invitation pack containing the study URL and a unique code to register if they would like to take part. After registering on the intervention website, participants will be asked to provide informed consent and complete screening and baseline measures.

Parents or legal representatives of potential participants for ECO-YP aged 13-15 years will be sent information about the study and a URL to provide online consent if they are happy for their child to take part. Upon receipt of parental consent, the 13-15-year-old will be sent a participant invitation pack with the intervention website URL and unique ID to sign up if they would like to take part. Once registered they will be asked to assent online.

Eligibility criteria

Eligibility for inclusion in ECO-YP: aged 13-25 years; identified from GP records as having eczema and have obtained a prescription for eczema treatment (emollient or topical corticosteroid) in the past 12 months; POEM score greater than five, to include mild to severe eczema, but exclude those with very mild or inactive eczema to avoid floor effects; have internet access.

Eligibility for inclusion in ECO-PC: parent / carer of a child aged 0-12 years; child identified from GP records as having eczema and has obtained a relevant prescription in the past 12 months; child has a POEM score greater than five, to include mild to severe eczema, but exclude those with very mild or inactive eczema; have internet access.

Only one person per household can take part in the trials. If a parent/carer has more than one child who meets the inclusion criteria they will be asked to specify one child to participate.

Potential participants from ECO-YP and ECO-PC are excluded if: unable to give informed consent; unable to read and write English, as the intervention content and outcome measures are in English; have taken part in another eczema study in the past 3-months; took part in think aloud interviews as part of ECO intervention development[15]. Qualitative interviewees who did not view intervention materials will not be excluded. See figure 1 for participant timeline.

For peer review only - http://bmjopen.bmj.com/site/about/guidelines.xhtml

Randomisation procedures and blinding

Participants will complete informed consent/assent and baseline questionnaires online within the intervention developed using LifeGuide software[16]. Those who do not meet the eligibility criteria of a minimum POEM score greater than 5 are presented with information explaining that they are not eligible for the study and signposted to other resources.

Eligible participants are randomised online to either 1) usual eczema care or 2) online intervention plus usual care through LifeGuide software. Randomisation is carried out in blocks and stratified by age (13-17; 18-25 (ECO-YP), and 0-5; 6-12 (ECO-PC), baseline eczema severity (POEM scores 6-7 (mild); 8-16 (moderate); 17-28 (severe) and recruitment region as these may influence how participants engage with the interventions.

It is not possible to mask participants to their allocation group. Participants are informed online as to which group they have been allocated to immediately after randomisation and are notified by email. The immediate trial team dealing with participant queries will have access to group allocation, but the wider Trial Management Group and trial statistician will remain blinded.

Intervention and group details

Usual care group

Participants randomised to usual care will continue to receive their usual medical advice and prescriptions. They can seek online support but will not be supported in doing so by the study team and will not have access to the online interventions during their participation in the trial. Participants allocated to the usual care group will be given access to the intervention after 52-week follow-up is complete.

Behavioural intervention groups (ECO-YP and ECO-PC)

Participants randomised to the intervention group will receive access to an online behavioural intervention to support eczema self-care in addition to usual eczema care, as above. The interventions were developed following the Person-Based Approach to intervention development [17,18] to ensure they are meaningful, optimally engaging, and relevant to target users, and draws on a theoretical framework including the Extended-Common Sense Model [19], Social Cognitive Theory [20], the Behaviour Change Wheel and associated Theoretical Domains Framework [21]. All intervention content is evidence-based, and the interventions are tailored and include interactive and audio-visual features. The interventions were initially developed by the research team consisting of behavioural psychologists, patient representatives, clinicians (GPs, dermatology nurse consultant, dermatologists), and skin researchers before being optimised through extensive user feedback to ensure they are acceptable, feasible, and optimally engaging to target users[22].

The online interventions target core behaviours linked to eczema management:

- regular use of emollients and appropriate use of topical corticosteroids
- avoiding eczema irritants and triggers
- minimising scratching
- emotional management.

The interventions use behavioural techniques to promote adherence and support eczema self-care by building on aspects like knowledge, skills, self-efficacy, social support, and environmental factors such as social and physical opportunity.

The interventions take participants through a core section before giving access to the main menu with the choice of various topics of interest to young people and families with eczema. These topics include eczema treatments, infections, talking to your healthcare professional, diet and allergy, sleep

For peer review only - http://bmjopen.bmj.com/site/about/guidelines.xhtml

and itch, physical activity, coping with stress, and transitioning to self-care. The interventions also include a 'two-week challenge' where participants are encouraged to use their eczema treatment regularly for two weeks, supported by optional text and email reminders and support. Intervention content has been developed to be interactive and engaging, with tailoring to suggest topics that may be of relevance. The intervention also contains a series of animated videos focussing on the core target behaviours.

ECO-YP has been developed for people aged 13 to 25 years with eczema. The intervention covers the topics mentioned above, as well as additional topics that are important particularly to this age group, such as information about finances, school / university /work, and cosmetics.

ECO-PC has been developed for parents of children aged 0 to 12 years with eczema. This intervention covers the same wide range of topics relevant to eczema, as well as sections that are specifically relevant to parents and co-management of eczema, such as transitioning to co-management, dealing with child resistance, and managing your child's eczema at nursery and school. Intervention description follows TIDieR guidelines[23]; detailed intervention development and optimisation studies will be published separately.

Outcomes

All participant reported outcome measures and intervention usage data are collected online, via LifeGuide software. Outcome measures are similar across ECO-YP and ECO-PC, where there are differences these are highlighted (Table 1). POEM, RECAP and itch intensity measures have been recommended as core outcome measures for eczema by the international Harmonising Outcome Measures for Eczema group [24,25].

Primary outcome

The primary outcome for both trials is the difference in patient-reported eczema severity between the intervention and usual care group as measured by POEM (Patient-Oriented Eczema Measure), every 4 weeks over 24 weeks [26,27].

POEM includes 7 questions about the frequency of eczema symptoms over the previous week that are summed to give a score from 0 (no eczema) to 28 (worst possible eczema). POEM can be completed by young people and children or by proxy (carer report, ECO-PC), demonstrates good validity, test-retest reliability and responsiveness to change [28].

Secondary outcomes

Secondary outcomes include: i) difference in POEM scores 4-weekly over 52 weeks; ii) Quality of Life at 24-weeks and 52-weeks, measured in ECO-YP, using the EQ-5D-5L[29] self-completed by the young person, and in ECO-PC by proxy using the Child Health Utility - Nine Dimensions (CHU-9D)[30] for children aged 2 to 12 years; iii) eczema control at 24-weeks and 52-weeks, measured by RECAP (Recap for atopic eczema patients)[31]; iv) itch intensity [32] at 24-weeks and 52-weeks, measured as worst itch in last 24 hours (not validated for proxy completion for children, and therefore used in ECO-YP only); v) patient enablement at 24-weeks and 52-weeks, the self-perceived ability to understand and cope with health issues, will be measured using the Patient Enablement Instrument (PEI) [33]; vi) health service use and medication use, measured by medical notes review for the 3-month period prior to baseline and the whole 52-week trial period; vii) cost-effectiveness combining quality of life and health service use and medication use.

Other measures

Prior belief about the effectiveness of the intervention and online resource use (websites or apps) for eczema will be measured at baseline and will be used in a planned subgroup analysis to explore

For peer review only - http://bmjopen.bmj.com/site/about/guidelines.xhtml

whether there is an interaction between prior belief, online resource use and treatment effectiveness.

Process measures

Self-reported barriers to adherence to eczema treatments will be measured at 24-weeks and 52-weeks using the Problematic Experiences of Therapy Scale (PETS)[34] and frequency of eczema treatment use (treatment adherence) will be measured by self-report. Intervention usage data for each participant will be automatically recorded by LifeGuide Software for the duration of the 52-week trial period.

Internal pilot phase

The first 3-months of participant recruitment was an internal pilot phase to test trial procedures, which mirrored the main trial protocol exactly. We assessed study uptake, recruitment and follow-up procedures, randomisation, and participant engagement in accessing the intervention. Success criteria for the pilot phase are listed in the full protocol (available from ECO website [14]).

Data collection methods and retention

All study procedures are automated and carried out online through the LifeGuide software[16]. Participants wishing to take part in the study provide consent and assent (where required) and complete an online baseline questionnaire before being randomised to either the usual care group or the intervention group. Participants in the intervention group then have access to the intervention website (either ECO-YP or ECO-PC).

All participants are asked to complete a 4-weekly POEM questionnaire online for 52 weeks. Participants are also asked to complete a longer 24-week and 52-week follow-up questionnaire online. When signing up for the trial participants are asked if they would prefer reminders by email, text message, or both. Automated emails and/or text messages are sent to notify participants when their follow-up questionnaires are available for completion. Reminders will be sent to non-responders after 5 days (and after 10 days for 24 and 52 week questionnaires), followed by reminder telephone calls approximately 4 days later from the research team, at which point participants will be invited to complete selected follow-up questions over the phone.

Sample size

The sample size calculation for ECO-YP and ECO-PC is based on 4-weekly POEM scores using repeated measures over the first 24 weeks of the trial, seeking to detect a minimum clinically important difference (MCID) of 2.5 points between groups (s.d. 6.5). Assuming a correlation between repeated measures of 0.70, with 90% power and 5% significance, this requires a total sample size of 121 per group in each of the two trials. Allowing for 20% loss to follow up gives a total sample size of 303 in each of the two trials. The sample size was amended during the trial, see protocol amendments section for details.

Statistical analysis plan

Primary analyses of trials ECO-YP and ECO-PC will be generalised linear mixed models, allowing for observations nested within participants over time. All analyses will control for key covariates, including age and baseline eczema severity, and will be set out in full in the Statistical Analysis Plan prior to database lock. For secondary outcome measures, linear models will be used for continuous outcomes. Where the assumptions for linear models are not met, we will use other appropriate distributions or non-parametric methods if no suitable distribution can be found. Logistic regression will be used for binary outcome measures.

For peer review only - http://bmjopen.bmj.com/site/about/guidelines.xhtml

We will collect data on use of other websites at the start and end of the trials to check whether there is a difference between groups in accessing other eczema sites and plan sensitivity analyses to examine whether accessing other resources affects outcomes. All trials of online interventions must assume that users in both groups may access other websites, and so trials provide a useful test of whether the intervention being evaluated is superior to the websites users can already access.

All analyses will be on an intention to treat basis (analysed as randomised), detailed in a Statistical Analysis Plan, and include participants from the internal pilots and full RCTs. No interim analyses are planned. The structure and pattern of missing data will be examined, if appropriate, and a sensitivity analysis based on data imputed using a multiple imputation model presented. Findings will be reported in accordance with the CONSORT statement.

Health economic evaluation

Two within trial economic evaluations will estimate whether ECO-YP and ECO-PC are cost-effective compared to usual care from an NHS and personal social services perspective. We will estimate the cost of the interventions and collect data on wider resource use (primary care, secondary care and accident and emergency use) and eczema-related prescriptions through medical notes review. Resource items will be valued using published unit costs for the most recent common price year to the time of analysis.

There is currently no agreed approach to valuing health outcomes in children in economic evaluations and there has been limited use of child and adolescent population-specific measures to generate health state utilities in NICE technology assessments.[35] In ECO-PC for parents/carers of children aged 0-12 we will collect by proxy the CHU-9D[36], a paediatric generic preference based instrument, in those aged 2 and over. Although the CHU-9D was developed for children aged 7 and over, its completion by proxy in younger age groups is currently being trialled [36] and the developer of the instrument has given us additional guidance to use with parents/carers with children in this age group. This approach is being taken as only parents/carers are expected to interact with the intervention.

In the ECO-YP (young people aged 13-25), all participants are asked to self-complete the EQ-5D-5L in order to estimate their health-related quality of life. To prevent any discontinuity, the EQ-5D-Y will not be used in those under the age of 16 as this is a different instrument to the EQ-5D-5L [37,38]. All participants will be asked to complete the EQ-5D-5L at baseline, 24 weeks and 52 weeks and the scores from these will be converted to utility scores using UK preference weights in line with current recommendations at the time of the analysis [37,39]. Following this, the utility values will be used to estimate Quality-Adjusted Life years (QALY) for the trial period using linear interpolation and area under the curve with and without baseline adjustment.[40]

Cost effectiveness (using change in POEM between baseline and 52 weeks, secondary analysis) and cost utility analyses (primary analysis) will be performed. Costs and benefits will not be discounted given the 12-month timeframe. Using information on costs and benefits, regression analysis will be conducted to estimate the incremental cost, incremental benefit and incremental cost utility of the online intervention compared to usual care (over the trial period). If one arm is clearly dominant (less costly and more effective) a recommendation can be made on this basis. If non-dominance occurs (that is if costs are greater and the intervention is more effective or if the intervention is cheaper and less effective), an incremental cost-effectiveness ratio (ICER) will be produced and a judgement about value for money will need to be made. The economic evaluation will be undertaken and analysed in line with guidelines.[41,42] Missing data will be dealt with in line with the approach taken in the main clinical statistical analysis, with sensitivity analysis undertaken to test the impact of approach if missing data is a particular problem. A detailed Health Economic Analysis Plan will be written and reviewed before the trial database is locked.

For peer review only - http://bmjopen.bmj.com/site/about/guidelines.xhtml

Nested process evaluation

The nested process evaluation studies are being carried out to understand intervention processes and participants' experiences of using the interventions.

Quantitative process evaluation

We will use baseline data to examine potential predictors and moderator effects of participant characteristics (e.g. age, eczema symptom severity, baseline attitudes) on intervention engagement (objectively recorded detailed website usage and self-reported treatment adherence) and outcome. We will also assess and analyse hypothesised mediators of treatment adherence and intervention outcomes; specifically changes in beliefs about treatment (PETS) as well as intervention usage. Objective measures of intervention usage are automatically recorded (with informed participant consent), allowing evaluations of usage patterns, such as time spent on intervention, number of visits to the intervention website, and pages visited.

Qualitative process evaluation

Qualitative process interviews will be carried out with approximately 30-40 participants (15 to 20 from ECO-YP and 15-20 from ECO-PC, or until saturation of the main themes are achieved). These interviews will provide in-depth understanding of patient and carers' experiences within the trial and provide a better understanding of factors that may influence engagement.

Interviews will be conducted via telephone or video call by a member of the research team experienced in qualitative research methods. We will interview participants from the intervention group and the usual care group and use purposive sampling to ensure a range of age, gender, ethnicity, eczema severity, website usage, deprivation index, and region. Potential participants will be contacted after being in the trial for at least 3 months by a member of the research team to check whether they would like to take part in an interview or have any questions about the study. Participants will be asked to give their consent online prior to the interview. Interviews will use a combination of open-ended and focussed questions and be transcribed verbatim.

Qualitative data will first be analysed using inductive thematic analysis.[43] We will then explore how emerging themes may map onto theoretical frameworks in order to relate our insights to generalisable theoretical constructs and inform implementation planning.

Process evaluation analysis

We will triangulate findings from the quantitative and qualitative process analyses [44] to explore and test the causal mechanisms proposed, to help inform interpretation of trial results, and determine how the interventions could be improved and how implementation into clinical practice could be facilitated.

Patient and Public Involvement (PPI)

The study team includes two PPI members (AR and AA) who have been involved in the project from the earliest stages. They are involved in all aspect of the ECO programme and trials, including intervention development[22], trial design, attending trial and programme management meetings, protocol discussions, developing participant facing materials, and co-authoring outputs. Our PPI partners will also be key for dissemination and future implementation.

ETHICS AND DISSEMINATION

For peer review only - http://bmjopen.bmj.com/site/about/guidelines.xhtml

The study has received the favourable opinion of South Central – Oxford A Research Ethics Committee (19/SC/0351). This summary protocol is based on approved protocol v3 (20/05/2020), ISRCTN reference 79282252.

Data monitoring

An independent Programme Steering Committee take responsibility for safeguarding the interest of study participants, monitor the main outcome measures including safety and efficacy, and monitor the overall conduct of the trials.

Protocol amendments

One substantial protocol amendment has been made since initial ethics approval (Substantial Amendment 1, number 47369.A4, approved 01/06/20). This amendment was in response to the Covid19 pandemic in order to:

1. Make all trial process online. The original protocol required parents or guardians of 13-15-year olds to return parental consent by post.
2. Increase in sample size. Our original sample size was 200 participants per trial, based on the published POEM MCID of 3[45]. However, research has since suggested that a smaller POEM MCID may be meaningful in certain contexts [46]. Recruitment to both trials exceeded expectation and a protocol amendment was made to change the sample size to a minimum of '200 participants' per trial to allow us to continue recruitment while a revised sample size was discussed with our Trial Management Group, Programme Management Group, Programme Steering Committee, and funder, without access to study outcome data or any interim analysis. The final agreed sample size for the trials were based on seeking to detect a POEM MCID of 2.5 points between groups, based on two repeated measures (s.d. 6.5), allowing for 20% loss to follow-up requiring sample size of 303 participants in each of the two trials.

Dissemination

As a minimum, study progress, outputs and trial findings will be made available via the study website[14] and project twitter (@ECO_eczema). Summaries will also be sent to participants and participating GP surgeries. Findings will be presented at conferences and published in peer-reviewed journals. We will make available a deidentified data set on request.

For peer review only - http://bmjopen.bmj.com/site/about/guidelines.xhtml

Figure 1. Flow of participants through the trial

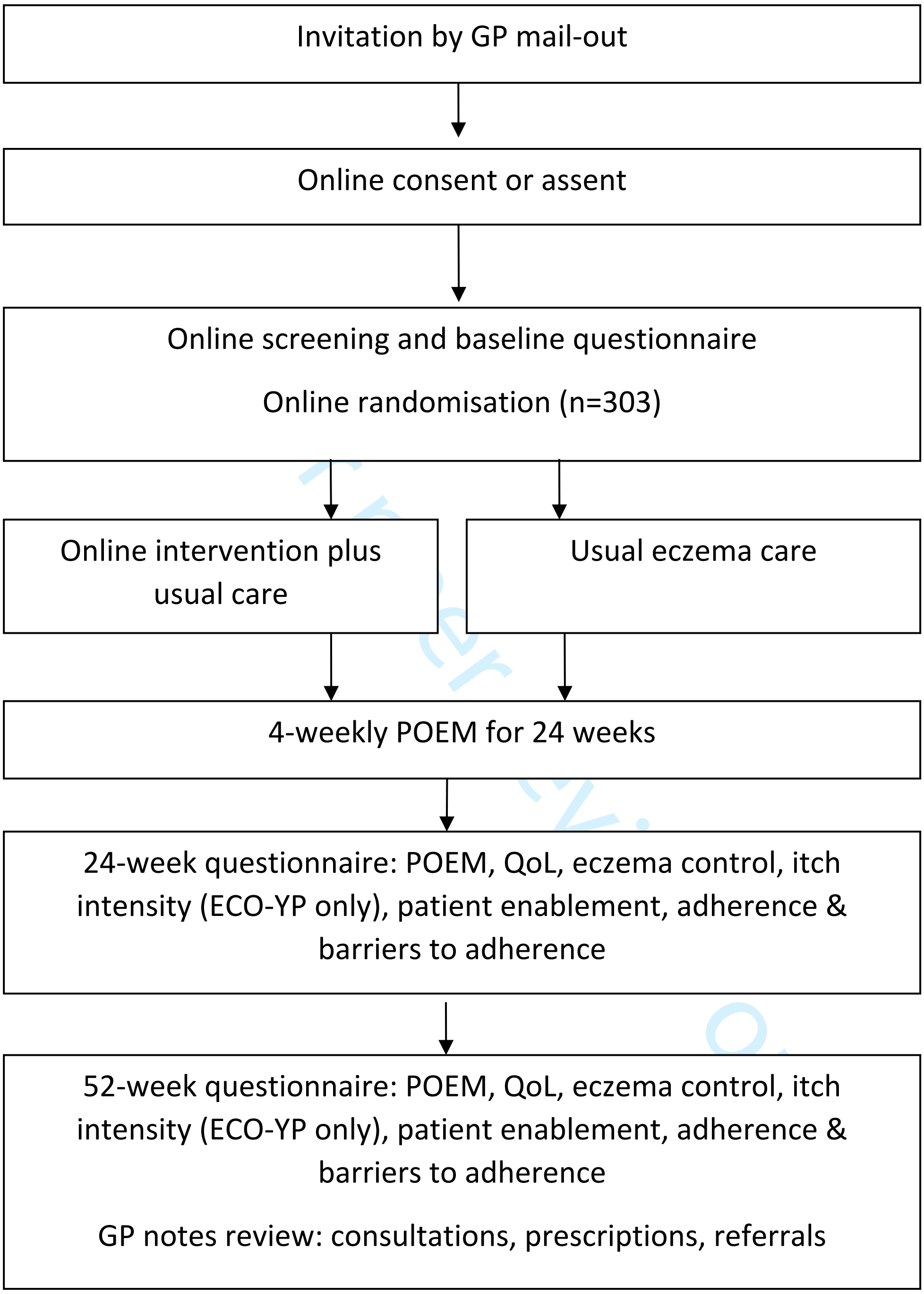

For peer review only - http://bmjopen.bmj.com/site/about/guidelines.xhtml

Table 1. Schedule of observations.

Outcomes collected	Baseline	24 weeks (primary outcome)	52 Weeks (end of study)
**Baseline characteristics**			
Demographics	✓		
Prior belief about effectiveness	✓		
Previous online resource use	✓		
**Clinical effectiveness outcomes**			
POEM (4-weekly)	✓	✓	✓
Long-term control (Recap)	✓	✓	✓
Itch intensity measure (ECO-YP only)	✓	✓	✓
Patient enablement Instrument (PEI)	✓	✓	✓
**Cost effectiveness outcomes**			
CHU-9D (ECO-PC for parents / carers of children aged 2-12 only)	✓	✓	✓
EQ-5D-5L (ECO-YP only)	✓	✓	✓
Medical notes review for medication use, service use, and referrals			✓ (including 3 months pre-baseline period)
**Process outcomes**			
Problematic Experiences of Therapy Scale (PETS)	✓	✓	✓
Frequency of eczema treatment use (adherence)	✓	✓	✓
Intervention usage			✓ (recorded throughout study)

For peer review only - http://bmjopen.bmj.com/site/about/guidelines.xhtml

AUTHOR CONTRIBUTIONS

MS and KT conceived the study idea and initial study design in collaboration with IM, LY, PLi, HW, JC, MR, SLa, BS, GG, TS, SiL, AR, AA, HK, with later input from JN, JH, SW, MSt, KG, KS. Specific advice was given by BS on trial design and medical statistics; IM, LY, KG, KS, PLe, LH on the process evaluation; and TS on the health economic evaluation. All the authors contributed to the drafting of the study protocol, led by IM, and approved the final manuscript.

FUNDING STATEMENT

This study presents independent research funded by the National Institute for Health Research (NIHR) under its Programme Grants for Applied Research programme (grant ref No RP-PG-0216-20007). Eczema Care Online (ECO) interventions were developed using LifeGuide software, which was partly funded by the NIHR Southampton Biomedical Research Centre (BRC). SiL was supported by a Wellcome senior research fellowship in clinical science (205039/Z/16/Z).

The views expressed are those of the author(s) and not necessarily those of the NHS, the NIHR or the Department of Health and Social Care.

COMPETING INTERESTS

No competing interests to declare

For peer review only

REFERENCES

1. Chamlin SL, Frieden IJ, Williams ML, et al. Effects of atopic dermatitis on young American children and their families. *Pediatrics* 2004;114(3):607-11.
2. Noerreslet M, Jemec GB, Traulsen JM. Involuntary autonomy: patients' perceptions of physicians, conventional medicines and risks in the management of atopic dermatitis. *Social Science & Medicine.69(9):1409-15* 2009.
3. Mollerup A, Johansen JD, Thing LF. Knowledge, attitudes and behaviour in everyday life with chronic hand eczema: a qualitative study. *Br J Dermatol* 2013;169(5):1056-65.
4. NICE. Clinical guideline 57. Atopic eczema in under 12s: diagnosis and management, 2007.
5. Bass AM, Anderson KL, Feldman SR. Interventions to Increase Treatment Adherence in Pediatric Atopic Dermatitis: A Systematic Review. *J Clin Med* 2015;4(2):231-42.
6. Beattie PE, Lewis-Jones MS. Parental knowledge of topical therapies in the treatment of childhood atopic dermatitis. *Clin Exp Dermatol* 2003;28:549-53.
7. Santer M, Burgess H, Yardley L, et al. Managing childhood eczema: qualitative study exploring carers' experiences of barriers and facilitators to treatment adherence. *J Adv Nurs* 2013;69(11):8.
8. Charman CR, Morris AD, Williams HC. Topical corticosteroid phobia in patients with atopic eczema. *British Journal of Dermatology* 2000;142(5):931-36.
9. Santer M, Burgess H, Yardley L, et al. Experiences of carers managing childhood eczema and their views on its treatment: a qualitative study. *British Journal of General Practice* 2012;62:261-67.
10. Powell K, Le Roux E, Banks J, et al. GP and parent dissonance about the assessment and treatment of childhood eczema in primary care: a qualitative study. *BMJ Open* 2018;8(2).
11. Horne R, Chapman SC, Parham R, et al. Understanding patients' adherence-related beliefs about medicines prescribed for long-term conditions: a meta-analytic review of the Necessity-Concerns Framework. *PLoS ONE [Electronic Resource]* 2013;8(12):e80633.
12. Santer M, Muller I, Yardley L, et al. 'You don't know which bits to believe': qualitative study exploring carers' experiences of seeking information on the internet about childhood eczema. *BMJ Open* 2015;5(4):e006339.
13. OfN S. Internet access – households and individuals, Great Britain: 2020. 2020.
14. ECO. *Eczema Care Online project website*. www.nottingham.ac.uk/eco (accessed 21/09/20).
15. Van den Haak MJ, De Jong MD, Schellens PJ. Evaluation of an informational web site: three variants of the think-aloud method compared. *Technical Communication* 2007;54(1):58-71.
16. Williams S, Yardley L, Weal M, et al. Introduction to LifeGuide: Open-source software for creating online interventions for health care, health promotion and training. *Knowledge Resources* 2010;187:90.
17. Yardley L, Morrison L, Bradbury K, et al. The person-based approach to intervention development: application to digital health-related behavior change interventions. *Journal of Medical Internet Research* 2015;17(1).
18. Morrison L, Muller I, Yardley L, et al. The person-based approach to planning, optimising, evaluating and implementing behavioural health interventions. *The European Health Psychologist* 2018;20(3):464-69.
19. Hagger MS, Orbell S. A meta-analytic review of the common-sense model of illness representations. *Psychology and health* 2003;18(2):141-84.
20. Bandura A. Self-efficacy: Towards a unifying theory and the organization. *Psychological Review* 1977;84:191-215.
21. Michie S, Van Stralen MM, West R. The behaviour change wheel: a new method for characterising and designing behaviour change interventions. *Implementation Science* 2011;6(1):42.

For peer review only - http://bmjopen.bmj.com/site/about/guidelines.xhtml

22. Muller I, Santer M, Morrison L, et al. Combining qualitative research with PPI: reflections on using the person-based approach for developing behavioural interventions. *Research Involvement and Engagement* 2019;5(1):34.
23. Hoffmann TC, Glasziou PP, Boutron I, et al. Better reporting of interventions: template for intervention description and replication (TIDieR) checklist and guide. *BMJ* 2014;348.
24. Spuls PI, Gerbens L, Simpson E, et al. Patient-Oriented Eczema Measure (POEM), a core instrument to measure symptoms in clinical trials: a Harmonising Outcome Measures for Eczema (HOME) statement. *British Journal of Dermatology* 2017;176(4):979-84.
25. CEBD. *Harmonising Outcome Measures for Eczema (HOME)*. http://www.homeforeczema.org (accessed 21/09/20).
26. Charman C, Venn AJ, Williams HC. The patient-oriented eczema measure: development and initial validation of a new tool for measuring atopic eczema severity from the patients' perspective. *Archives of dermatology* 2004;140 (12):1513-19.
27. Charman C, Venn AJ, Ravenscroft JC, et al. Translating Patient-Oriented Eczema Measure (POEM) scores into clinical practice by suggesting severity strata derived using anchor-based methods, 2013:1326-32.
28. Schmitt J, Langan S, Deckert S, et al. Assessment of clinical signs of atopic dermatitis: A systematic review and recommendation. *Journal of Allergy and Clinical Immunology* 2013;132(6):1337-47.
29. Herdman M, Gudex C, Lloyd A, et al. Development and preliminary testing of the new five-level version of EQ-5D (EQ-5D-5L). *Qual Life Res* 2011;20(10):1727-36.
30. Stevens K. Valuation of the child health utility 9D index. *Pharmacoeconomics* 2012;30(8):729-47.
31. Howells L, Chalmers J, Gran S, et al. Development and initial testing of a new instrument to measure the experience of eczema control in adults and children: Recap of atopic eczema (RECAP). *British Journal of Dermatology* 2020;183(3):524-36.
32. Yosipovitch G, Reaney M, Mastey V, et al. Peak Pruritus Numerical Rating Scale: psychometric validation and responder definition for assessing itch in moderate-to-severe atopic dermatitis. *British Journal of Dermatology* 2019;181(4):761-69.
33. Howie JGR, Heaney DJ, M. M, et al. A Comparison of a Patinet Enablement Intrument (PEI) Against Two Established Satisfaciton Scales as an Outcome Measure of Primary Care Consultation. *Family Practice* 1998;15(2):165-71.
34. Kirby S, Donovan-Hall M, Yardley L. Measuring barriers to adherence: validation of the Problematic Experiences of Therapy Scale. *Disabil Rehabil* 2014;36(22):1924-9.
35. Ungar W. *Economic evaluation in child health*: Oxford University Press; 2009.
36. Canaway AG, Frew EJ. Measuring preference-based quality of life in children aged 6-7 years: a comparison of the performance of the CHU-9D and EQ-5D-Y--the WAVES pilot study. *Qual Life Res* 2013;22(1):173-83.
37. Devlin N SK, Feng Y, Mulhern B. van Hout, B. *Valuing Health-Related Quality of Life: An EQ-5D-5L Value Set for England*. https://www.sheffield.ac.uk/scharr/sections/heds/discussion-papers/16-02-1.546901 (accessed 6 April).
38. EuroQOL. *EQ-5D-Y User Guide V1.0*. https://euroqol.org/wp-content/uploads/2016/09/EQ-5D-Y_User_Guide_v1.0_2014.pdf (accessed 13 December).
39. NICE. *Position statement on use of the EQ-5D-5L valuation set.* . https://www.nice.org.uk/Media/Default/About/what-we-do/NICE-guidance/NICE-technology-appraisal-guidance/eq5d5l_nice_position_statement.pdf (accessed 20 August).
40. Manca A, Hawkins N, Sculpher MJ. Estimating mean QALYs in trial-based cost-effectiveness analysis: the importance of controlling for baseline utility. *Health Econ* 2005;14(5):487-96.
41. Ramsey SD, Willke RJ, Glick H, et al. Cost-Effectiveness Analysis Alongside Clinical Trials II—An ISPOR Good Research Practices Task Force Report. *Value in Health* 2015;18(2):161-72.
42. Drummond MF, Sculpher M, Claxton K, et al. *Methods for the economic evaluation of health care programmes*. 4th edition ed. Oxford: Oxford University Press; 2015.

For peer review only - http://bmjopen.bmj.com/site/about/guidelines.xhtml

43. Braun V, Clarke V. Using thematic analysis in psychology. *Qualitative Research in Psychology* 2006;3(2):77-101.
44. Yardley L, Bishop F. Mixing qualitative and quantitative methods: A pragmatic approach. In: Willig C, Stainton-Rogers W (eds.) *Qualitative Research in Psychology*. Los Angeles, USA: Sage; 2007 p313-26.
45. Schram ME, Spuls PI, Leeflang MM, et al. EASI, (objective) SCORAD and POEM for atopic eczema: responsiveness and minimal clinically important difference. *Allergy* 2012;67(1):99-106.
46. Howells L, Ratib S, Chalmers J, et al. How should minimally important change scores for the Patient-Oriented Eczema Measure be interpreted? A validation using varied methods. *British Journal of Dermatology* 2018;178(5):1135-42.

For peer review only

For peer review only - http://bmjopen.bmj.com/site/about/guidelines.xhtml

SPIRIT

STANDARD PROTOCOL ITEMS: RECOMMENDATIONS FOR INTERVENTIONAL TRIALS

SPIRIT 2013 Checklist: Recommended items to address in a clinical trial protocol and related documents*

Section/item	Item No	Description	Addressed on page number
**Administrative information**			
Title	1	Descriptive title identifying the study design, population, interventions, and, if applicable, trial acronym	___1_________
Trial registration	2a	Trial identifier and registry name. If not yet registered, name of intended registry	___2_________
	2b	All items from the World Health Organization Trial Registration Data Set	See protocol on website
Protocol version	3	Date and version identifier	See protocol on website
Funding	4	Sources and types of financial, material, and other support	_____13________
Roles and responsibilities	5a	Names, affiliations, and roles of protocol contributors	_____1 & 13________
	5b	Name and contact information for the trial sponsor	See protocol on website
	5c	Role of study sponsor and funders, if any, in study design; collection, management, analysis, and interpretation of data; writing of the report; and the decision to submit the report for publication, including whether they will have ultimate authority over any of these activities	See protocol on website
	5d	Composition, roles, and responsibilities of the coordinating centre, steering committee, endpoint adjudication committee, data management team, and other individuals or groups overseeing the trial, if applicable (see Item 21a for data monitoring committee)	_____10________

For peer review only

Introduction

Background and rationale	6a	Description of research question and justification for undertaking the trial, including summary of relevant studies (published and unpublished) examining benefits and harms for each intervention	__3__________
	6b	Explanation for choice of comparators	__3__________
Objectives	7	Specific objectives or hypotheses	__3__________
Trial design	8	Description of trial design including type of trial (eg, parallel group, crossover, factorial, single group), allocation ratio, and framework (eg, superiority, equivalence, noninferiority, exploratory)	__3__________

Methods: Participants, interventions, and outcomes

Study setting	9	Description of study settings (eg, community clinic, academic hospital) and list of countries where data will be collected. Reference to where list of study sites can be obtained	__4__________
Eligibility criteria	10	Inclusion and exclusion criteria for participants. If applicable, eligibility criteria for study centres and individuals who will perform the interventions (eg, surgeons, psychotherapists)	__4__________
Interventions	11a	Interventions for each group with sufficient detail to allow replication, including how and when they will be administered	__5__________
	11b	Criteria for discontinuing or modifying allocated interventions for a given trial participant (eg, drug dose change in response to harms, participant request, or improving/worsening disease)	__n/a__________
	11c	Strategies to improve adherence to intervention protocols, and any procedures for monitoring adherence (eg, drug tablet return, laboratory tests)	__6__________
	11d	Relevant concomitant care and interventions that are permitted or prohibited during the trial	__5__________
Outcomes	12	Primary, secondary, and other outcomes, including the specific measurement variable (eg, systolic blood pressure), analysis metric (eg, change from baseline, final value, time to event), method of aggregation (eg, median, proportion), and time point for each outcome. Explanation of the clinical relevance of chosen efficacy and harm outcomes is strongly recommended	__6__________
Participant timeline	13	Time schedule of enrolment, interventions (including any run-ins and washouts), assessments, and visits for participants. A schematic diagram is highly recommended (see Figure)	Figure 1, p11

For peer review only - http://bmjopen.bmj.com/site/about/guidelines.xhtml

Sample size	14	Estimated number of participants needed to achieve study objectives and how it was determined, including clinical and statistical assumptions supporting any sample size calculations	___8__________
Recruitment	15	Strategies for achieving adequate participant enrolment to reach target sample size	___7_________
**Methods: Assignment of interventions (for controlled trials)**			
Allocation:			
Sequence generation	16a	Method of generating the allocation sequence (eg, computer-generated random numbers), and list of any factors for stratification. To reduce predictability of a random sequence, details of any planned restriction (eg, blocking) should be provided in a separate document that is unavailable to those who enrol participants or assign interventions	___5_________
Allocation concealment mechanism	16b	Mechanism of implementing the allocation sequence (eg, central telephone; sequentially numbered, opaque, sealed envelopes), describing any steps to conceal the sequence until interventions are assigned	___5_________
Implementation	16c	Who will generate the allocation sequence, who will enrol participants, and who will assign participants to interventions	___5_________
Blinding (masking)	17a	Who will be blinded after assignment to interventions (eg, trial participants, care providers, outcome assessors, data analysts), and how	___5______
	17b	If blinded, circumstances under which unblinding is permissible, and procedure for revealing a participant's allocated intervention during the trial	Not applicable
**Methods: Data collection, management, and analysis**			
Data collection methods	18a	Plans for assessment and collection of outcome, baseline, and other trial data, including any related processes to promote data quality (eg, duplicate measurements, training of assessors) and a description of study instruments (eg, questionnaires, laboratory tests) along with their reliability and validity, if known. Reference to where data collection forms can be found, if not in the protocol	____7__________
	18b	Plans to promote participant retention and complete follow-up, including list of any outcome data to be collected for participants who discontinue or deviate from intervention protocols	____7________

Data management	19	Plans for data entry, coding, security, and storage, including any related processes to promote data quality (eg, double data entry; range checks for data values). Reference to where details of data management procedures can be found, if not in the protocol	____7________
Statistical methods	20a	Statistical methods for analysing primary and secondary outcomes. Reference to where other details of the statistical analysis plan can be found, if not in the protocol	____8________
	20b	Methods for any additional analyses (eg, subgroup and adjusted analyses)	____8________
	20c	Definition of analysis population relating to protocol non-adherence (eg, as randomised analysis), and any statistical methods to handle missing data (eg, multiple imputation)	____8________
**Methods: Monitoring**			
Data monitoring	21a	Composition of data monitoring committee (DMC); summary of its role and reporting structure; statement of whether it is independent from the sponsor and competing interests; and reference to where further details about its charter can be found, if not in the protocol. Alternatively, an explanation of why a DMC is not needed	____10_______
	21b	Description of any interim analyses and stopping guidelines, including who will have access to these interim results and make the final decision to terminate the trial	___7_________
Harms	22	Plans for collecting, assessing, reporting, and managing solicited and spontaneously reported adverse events and other unintended effects of trial interventions or trial conduct	See protocol on website
Auditing	23	Frequency and procedures for auditing trial conduct, if any, and whether the process will be independent from investigators and the sponsor	See protocol on website
**Ethics and dissemination**			
Research ethics approval	24	Plans for seeking research ethics committee/institutional review board (REC/IRB) approval	____9________
Protocol amendments	25	Plans for communicating important protocol modifications (eg, changes to eligibility criteria, outcomes, analyses) to relevant parties (eg, investigators, REC/IRBs, trial participants, trial registries, journals, regulators)	____10_______

Consent or assent	26a	Who will obtain informed consent or assent from potential trial participants or authorised surrogates, and how (see Item 32)	____4_______
	26b	Additional consent provisions for collection and use of participant data and biological specimens in ancillary studies, if applicable	Not applicable
Confidentiality	27	How personal information about potential and enrolled participants will be collected, shared, and maintained in order to protect confidentiality before, during, and after the trial	See protocol on website
Declaration of interests	28	Financial and other competing interests for principal investigators for the overall trial and each study site	___13________
Access to data	29	Statement of who will have access to the final trial dataset, and disclosure of contractual agreements that limit such access for investigators	_____________
Ancillary and post-trial care	30	Provisions, if any, for ancillary and post-trial care, and for compensation to those who suffer harm from trial participation	_____________
Dissemination policy	31a	Plans for investigators and sponsor to communicate trial results to participants, healthcare professionals, the public, and other relevant groups (eg, via publication, reporting in results databases, or other data sharing arrangements), including any publication restrictions	____10_______
	31b	Authorship eligibility guidelines and any intended use of professional writers	See protocol on website
	31c	Plans, if any, for granting public access to the full protocol, participant-level dataset, and statistical code	Full protocol is on study website.
**Appendices**			
Informed consent materials	32	Model consent form and other related documentation given to participants and authorised surrogates	_____________
Biological specimens	33	Plans for collection, laboratory evaluation, and storage of biological specimens for genetic or molecular analysis in the current trial and for future use in ancillary studies, if applicable	_____________

*It is strongly recommended that this checklist be read in conjunction with the SPIRIT 2013 Explanation & Elaboration for important clarification on the items. Amendments to the protocol should be tracked and dated. The SPIRIT checklist is copyrighted by the SPIRIT Group under the Creative Commons "Attribution-NonCommercial-NoDerivs 3.0 Unported" license.

For peer review only - http://bmjopen.bmj.com/site/about/guidelines.xhtml

BMJ Open

Supporting self-care for eczema: protocol for two randomised controlled trials of ECO (Eczema Care Online) interventions for young people and parents/carers

Journal:	*BMJ Open*
Manuscript ID	bmjopen-2020-045583.R1
Article Type:	Protocol
Date Submitted by the Author:	22-Dec-2020

Complete List of Authors:	Muller, Ingrid; University of Southampton, School of Primary Care, Population Health and Medical Education<br>Stuart, Beth; University of Southampton, School of Primary Care, Population Health and Medical Education<br>Sach, Tracey; University of East Anglia, Norwich Medical School<br>Hooper, Julie; University of Southampton, School of Primary Care, Population Health and Medical Education<br>Wilczynska, Sylvia; University of Southampton, School of Primary Care, Population Health and Medical Education,<br>Steele, Mary; University of Southampton School of Psychology,<br>Greenwell, Kate; University of Southampton, Department of Psychology<br>Sivyer, Katy; University of Southampton, Department of Psychology; University of Portsmouth, Department of Psychology<br>Yardley, Lucy; University of Southampton, Department of Psychology; University of Bristol, School of Experimental Psychology<br>Williams, HC; University of Nottingham, Centre of Evidence Based Dermatology<br>Chalmers, Joanne; University of Nottingham, Centre of Evidence Based Dermatology<br>Leighton, Paul; University of Nottingham, Centre of Evidence Based Dermatology<br>Howells, Laura; University of Nottingham Centre for Evidence Based Dermatology,<br>Ridd, Matthew; University of Bristol Faculty of Health Sciences, Population Health Sciences;<br>Lawton, Sandra; Rotherham NHS Foundation Trust, Dermatology Department<br>Griffiths, Gareth; University of Southampton, Southampton Clinical Trials Unit<br>Nuttall, Jacqui; University of Southampton, Southampton Clinical Trials Unit<br>Langan, Sinead; London School of Hygiene & Tropical Medicine,<br>Roberts, Amanda; University of Nottingham, Centre of Evidence-Based Dermatology<br>Ahmed, Amina; University of Nottingham, Centre of Evidence Based Dermatology<br>Kirk, Hayden; Solent NHS Trust, Neurological Rehabilitation<br>Becque, Taeko; University of Southampton, Primary Care and Population Sciences

	Little, Paul; University of Southampton, School of Primary Care, Population Health and Medical Education<br>Thomas, Kim; University of Nottingham, Centre of Evidence Based Dermatology<br>Santer, Miriam; University of Southampton, School of Primary Care, Population Health and Medical Education
**Primary Subject Heading**:	General practice / Family practice
Secondary Subject Heading:	Dermatology
Keywords:	Eczema < DERMATOLOGY, PRIMARY CARE, World Wide Web technology < BIOTECHNOLOGY & BIOINFORMATICS

SCHOLARONE™
Manuscripts

I, the Submitting Author has the right to grant and does grant on behalf of all authors of the Work (as defined in the below author licence), an exclusive licence and/or a non-exclusive licence for contributions from authors who are: i) UK Crown employees; ii) where BMJ has agreed a CC-BY licence shall apply, and/or iii) in accordance with the terms applicable for US Federal Government officers or employees acting as part of their official duties; on a worldwide, perpetual, irrevocable, royalty-free basis to BMJ Publishing Group Ltd ("BMJ") its licensees and where the relevant Journal is co-owned by BMJ to the co-owners of the Journal, to publish the Work in this journal and any other BMJ products and to exploit all rights, as set out in our licence.

The Submitting Author accepts and understands that any supply made under these terms is made by BMJ to the Submitting Author unless you are acting as an employee on behalf of your employer or a postgraduate student of an affiliated institution which is paying any applicable article publishing charge ("APC") for Open Access articles. Where the Submitting Author wishes to make the Work available on an Open Access basis (and intends to pay the relevant APC), the terms of reuse of such Open Access shall be governed by a Creative Commons licence – details of these licences and which Creative Commons licence will apply to this Work are set out in our licence referred to above.

Other than as permitted in any relevant BMJ Author's Self Archiving Policies, I confirm this Work has not been accepted for publication elsewhere, is not being considered for publication elsewhere and does not duplicate material already published. I confirm all authors consent to publication of this Work and authorise the granting of this licence.

For peer review only

Supporting self-care for eczema: protocol for two randomised controlled trials of ECO (Eczema Care Online) interventions for young people and parents/carers

Ingrid Muller[1], Beth Stuart[1], Tracey H Sach[2], Julie Hooper[1], Sylvia Wilczynska[1], Mary Steele[1], Kate Greenwell[3], Katy Sivyer[34], Lucy Yardley[3, 5], HC Williams[6], Joanne R Chalmers[6], Paul Leighton[6], Laura Howells[6], Matthew J Ridd[7], Sandra Lawton[8], Gareth Griffiths[9], Jacqui Nuttall[9], Sinéad M Langan[10], Amanda Roberts[11], Amina Ahmed[6], Hayden Kirk[12], Taeko Becque[1], Paul little[1], Kim Suzanne Thomas[6], Miriam Santer[1]

[1] School of Primary Care, Population Health and Medical Education, University of Southampton, UK

[2] Norwich Medical School, University of East Anglia, Norfolk, UK

[3] Centre for Clinical and Community Applications of Health Psychology, University of Southampton, Southampton, UK

[4] Department of Psychology, Faculty of Science and Health, University of Portsmouth, UK

[5] School of Experimental Psychology, University of Bristol, Bristol, UK

[6] Centre of Evidence Based Dermatology, School of Medicine, University of Nottingham, Nottingham, UK

[7] Population Health Sciences, University of Bristol, Bristol, UK

[8] Rotherham NHS Foundation Trust, Rotherham, UK

[9] Southampton Clinical Trials Unit, University of Southampton, Southampton, UK

[10] Faculty of Epidemiology and Population Health, London School of Hygiene and Tropical Medicine, London, UK

[11] Nottingham Support Group for Carers of Children with Eczema, Nottingham, UK

[12] Solent NHS Trust, Southampton, UK

Word count: 3992

Keywords: Atopic eczema / dermatitis, behavioural intervention, RCT, online, self-management, cost-effectiveness

Corresponding author:

Ingrid Muller

School of Primary Care, Population Health and Medical Education, University of Southampton, UK

I.Muller@soton.ac.uk

For peer review only - http://bmjopen.bmj.com/site/about/guidelines.xhtml

Abstract

Introduction

Eczema care requires management of triggers and various treatments. We developed two online behavioural interventions to support eczema care called ECO (Eczema Care Online) for young people and ECO for families. This protocol describes two randomised controlled trials aimed to evaluate clinical and cost effectiveness of the two interventions.

Methods and analysis

Design: Two independent, pragmatic, unmasked, parallel group randomised controlled trials with internal pilots and nested health economic and process evaluation studies. *Setting*: Participants will be recruited from GP practices in England. *Participants*: young people aged 13-25 years with eczema and parents / carers of children aged 0-12 years with eczema, excluding inactive or very mild eczema (5 or less on Patient-Oriented Eczema Measure (POEM)). *Interventions*: Participants will be randomised to online intervention plus usual care or to usual eczema care alone. *Outcome measures*: Primary outcome is eczema severity over 24 weeks measured by POEM. Secondary outcomes include: POEM 4-weekly for 52 weeks, quality of life, eczema control, itch intensity (young people only), patient enablement, health service and treatment use. Process measures include treatment adherence, barriers to adherence, and intervention usage. Our sample sizes of 303 participants per trial are powered to detect a group difference of 2.5 (SD 6.5) in monthly POEM scores over 24 weeks (significance 0.05, power 0.9), allowing for 20% loss to follow-up. Cost effectiveness analysis will be from an NHS and personal social service perspective. Qualitative and quantitative process evaluation will help understand mechanisms of action and participant experiences and inform implementation.

Ethics and dissemination

The study has been approved by South Central Oxford A Research Ethics Committee (19/SC/0351). Recruitment is ongoing, and follow-up will be completed by mid-2022. Findings will be disseminated to participants, the public, dermatology and primary care journals, and policymakers.

Trial registration number: ISRCTN79282252

ARTICLE SUMMARY

Strengths and limitations of this study

- Two large RCTs of online complex behavioural interventions addressing an important clinical need and research gap to support eczema self-care.
- Comprehensive intervention development following the Person-Based Approach with extensive input from young people and families with eczema.
- Both trials include qualitative and quantitative process evaluation to understand the interventions' mechanisms of action and participant experiences.
- Cost-effectiveness of both interventions will be evaluated in nested health economic studies.
- Our primary outcome is self-reported eczema severity using the Patient-Oriented Eczema Measure (POEM), but the lack of assessment of objective eczema severity could be viewed as a limitation.

For peer review only - http://bmjopen.bmj.com/site/about/guidelines.xhtml

For peer review only

INTRODUCTION

Background and rationale

Eczema can cause substantial impact on quality of life, primarily because of sleep disturbance and itch.[1] Families of children with eczema express frustration that they do not receive enough information about how to manage the condition,[2] as do adults with eczema.[3] NICE guidance on eczema [4] highlights that the main cause of treatment failure is non-adherence and there is a need for new ways to support adherence.[5] Reasons for non-adherence include therapy being time-intensive [6,7], lack of understanding of treatments and how to use them [6], under-use of topical corticosteroids related to concerns about side-effects [8], conflicting advice from different health professionals regarding how to use topical corticosteroids[9,10], and child-refusal[7].

Self-care includes all the health behaviours needed to look after one's own condition. Non-adherence is related to people's understanding of their condition and its treatment, as well as perceived need for treatments and concerns about adverse consequences of treatments.[11] Self-care is particularly complex in eczema as it involves regular application of topical treatments (mainly emollients for maintenance and topical corticosteroids for inflamed eczema) and avoidance of triggers (e.g. soap). Presently, many people / families receive little advice on how to manage the condition, or obtain advice of variable quality from the internet.[12] There is a need for high quality, accessible interventions, as well as evidence of whether interventions work so that, if effective, clinicians can signpost towards these as an essential part of routine care.

Currently, 96% of British households have access to the internet with 99% of adults being regular internet users.[13] Although information about eczema is widely available on the internet, it is of variable quality, often promoting commercial products of unproven efficacy. Patients and parents/carers find it difficult to know which information is reliable.[12]

We have developed two web-based interventions to support eczema management; ECO (Eczema Care Online) for parents and carers of children aged 0-12 years with eczema, and ECO for young people aged 13-25 years with eczema. Parents of children with eczema and young people with eczema are likely to have different support and information needs. We have therefore developed two separate interventions to be evaluated in two separate RCTs. This paper provides an abridged version of the full protocol that is available on the project website[14].

Study objectives

The primary objective is to determine the clinical effectiveness of two online interventions compared to usual care for eczema: one for young people aged 13-25 with eczema (ECO-YP) and one for parents/carers of children aged 0-12 with eczema (ECO-PC).

Secondary objectives are: i) to determine the cost effectiveness of the online interventions from a National Health Service (NHS) and personal social service perspective, and ii) to determine the interventions' mechanisms of action and factors related to participant engagement and treatment adherence and its outcomes.

Trial design

This protocol comprises two independent pragmatic, parallel group 1:1 allocation individually randomised superiority trials:

For peer review only - http://bmjopen.bmj.com/site/about/guidelines.xhtml

1. ECO-YP: to assess the effectiveness of an online intervention in young people (YP) with eczema aged 13-25 years as measured by Patient-Oriented Eczema Measure (POEM) 4-weekly scores over 24 weeks.
2. ECO-PC: to assess the effectiveness of an online intervention in parents and carers (PC) of children with eczema aged 0-12 years as measured by Patient-Oriented Eczema Measure (POEM) 4-weekly scores over 24 weeks.

Total duration of follow-up will be 52 weeks with primary outcome assessed over the first 24 weeks.

METHODS AND ANALYSIS

Study setting

Primary care (General practitioner (GP) surgeries) in Wessex, West of England, East Midlands, and Thames Valley and South Midlands.

Recruitment

We will identify children with eczema aged 0-12 years and young people with eczema aged 13-25 years via an electronic records search developed by the study team and run by staff at the participating GP surgeries. A doctor or delegated member of the practice team will screen the identified list to assess suitability to receive a study invitation. Potential participants will be sent an invitation pack containing the study URL and a unique code to register if they would like to take part. After registering on the intervention website, participants will be asked to provide informed consent and complete screening and baseline measures.

Parents or legal representatives of potential participants for ECO-YP aged 13-15 years will be sent information about the study and a URL to provide online consent if they are happy for their child to take part. Upon receipt of parental consent, the 13-15-year-old will be sent a participant invitation pack with the intervention website URL and unique ID to sign up if they would like to take part. Once registered they will be asked to assent online.

Eligibility criteria

Eligibility for inclusion in ECO-YP: aged 13-25 years; identified from GP records as having eczema and have obtained a prescription for eczema treatment (emollient or topical corticosteroid) in the past 12 months; POEM score greater than five, to include mild to severe eczema, but exclude those with very mild or inactive eczema to avoid floor effects; have internet access.

Eligibility for inclusion in ECO-PC: parent / carer of a child aged 0-12 years; child identified from GP records as having eczema and has obtained a relevant prescription in the past 12 months; child has a POEM score greater than five, to include mild to severe eczema, but exclude those with very mild or inactive eczema; have internet access.

Only one person per household can take part in the trials. If a parent/carer has more than one child who meets the inclusion criteria they will be asked to specify one child to participate.

Potential participants from ECO-YP and ECO-PC are excluded if: unable to give informed consent; unable to read and write English, as the intervention content and outcome measures are in English; have taken part in another eczema study in the past 3-months; took part in think aloud interviews as part of ECO intervention development[15]. Qualitative interviewees who did not view intervention materials will not be excluded. See figure 1 for participant timeline.

For peer review only - http://bmjopen.bmj.com/site/about/guidelines.xhtml

Randomisation procedures and blinding

Participants will complete informed consent/assent and baseline questionnaires online within the intervention developed using LifeGuide software[16]. Those who do not meet the eligibility criteria of a minimum POEM score greater than 5 are presented with information explaining that they are not eligible for the study and signposted to other resources.

Eligible participants are randomised online to either 1) usual eczema care or 2) online intervention plus usual care through LifeGuide software. Randomisation is carried out in blocks and stratified by age (13-17; 18-25 (ECO-YP), and 0-5; 6-12 (ECO-PC), baseline eczema severity (POEM scores 6-7 (mild); 8-16 (moderate); 17-28 (severe) and recruitment region as these may influence how participants engage with the interventions.

It is not possible to mask participants to their allocation group. Participants are informed online as to which group they have been allocated to immediately after randomisation and are notified by email. The immediate trial team dealing with participant queries will have access to group allocation, but the wider Trial Management Group and trial statistician will remain blinded.

Intervention and group details

Usual care group

Participants randomised to usual care will continue to receive their usual medical advice and prescriptions. They can seek online support but will not be supported in doing so by the study team and will not have access to the online interventions during their participation in the trial. Participants allocated to the usual care group will be given access to the intervention after 52-week follow-up is complete.

Behavioural intervention groups (ECO-YP and ECO-PC)

Participants randomised to the intervention group will receive access to an online behavioural intervention to support eczema self-care in addition to usual eczema care, as above. The interventions were developed following the Person-Based Approach to intervention development [17,18] to ensure they are meaningful, optimally engaging, and relevant to target users, and draws on a theoretical framework including the Extended-Common Sense Model [19], Social Cognitive Theory [20], the Behaviour Change Wheel and associated Theoretical Domains Framework [21]. All intervention content is evidence-based, and the interventions are tailored and include interactive and audio-visual features. The interventions were initially developed by the research team consisting of behavioural psychologists, patient representatives, clinicians (GPs, dermatology nurse consultant, dermatologists), and skin researchers before being optimised through extensive user feedback to ensure they are acceptable, feasible, and optimally engaging to target users[22].

The online interventions target core behaviours linked to eczema management:

- regular use of emollients and appropriate use of topical corticosteroids
- avoiding eczema irritants and triggers
- minimising scratching
- emotional management.

The interventions use behavioural techniques to promote adherence and support eczema self-care by building on aspects like knowledge, skills, self-efficacy, social support, and environmental factors such as social and physical opportunity.

The interventions take participants through a core section before giving access to the main menu with the choice of various topics of interest to young people and families with eczema. These topics include eczema treatments, infections, talking to your healthcare professional, diet and allergy, sleep

For peer review only - http://bmjopen.bmj.com/site/about/guidelines.xhtml

and itch, physical activity, coping with stress, and transitioning to self-care. The interventions also include a 'two-week challenge' where participants are encouraged to use their eczema treatment regularly for two weeks, supported by optional text and email reminders and support. Intervention content has been developed to be interactive and engaging, with tailoring to suggest topics that may be of relevance. The intervention also contains a series of animated videos focussing on the core target behaviours.

ECO-YP has been developed for people aged 13 to 25 years with eczema. The intervention covers the topics mentioned above, as well as additional topics that are important particularly to this age group, such as information about finances, school / university /work, and cosmetics.

ECO-PC has been developed for parents of children aged 0 to 12 years with eczema. This intervention covers the same wide range of topics relevant to eczema, as well as sections that are specifically relevant to parents and co-management of eczema, such as transitioning to co-management, dealing with child resistance, and managing your child's eczema at nursery and school. Intervention description follows TIDieR guidelines[23]; detailed intervention development and optimisation studies will be published separately.

Outcomes

All participant reported outcome measures and intervention usage data are collected online, via LifeGuide software. Outcome measures are similar across ECO-YP and ECO-PC, where there are differences these are highlighted (Table 1). POEM, RECAP and itch intensity measures have been recommended as core outcome measures for eczema by the international Harmonising Outcome Measures for Eczema group [24,25].

Primary outcome

The primary outcome for both trials is the difference in patient-reported eczema severity between the intervention and usual care group as measured by POEM (Patient-Oriented Eczema Measure), every 4 weeks over 24 weeks [26,27].

POEM includes 7 questions about the frequency of eczema symptoms over the previous week that are summed to give a score from 0 (no eczema) to 28 (worst possible eczema). POEM can be completed by young people and children or by proxy (carer report, ECO-PC), demonstrates good validity, test-retest reliability and responsiveness to change [28].

Secondary outcomes

Secondary outcomes include: i) difference in POEM scores 4-weekly over 52 weeks; ii) Quality of Life at 24-weeks and 52-weeks, measured in ECO-YP, using the EQ-5D-5L[29] self-completed by the young person, and in ECO-PC by proxy using the Child Health Utility - Nine Dimensions (CHU-9D)[30] for children aged 2 to 12 years; iii) eczema control at 24-weeks and 52-weeks, measured by RECAP (Recap for atopic eczema patients)[31]; iv) itch intensity [32] at 24-weeks and 52-weeks, measured as worst itch in last 24 hours (not validated for proxy completion for children, and therefore used in ECO-YP only); v) patient enablement at 24-weeks and 52-weeks, the self-perceived ability to understand and cope with health issues, will be measured using the Patient Enablement Instrument (PEI) [33]; vi) health service use and medication use, measured by medical notes review for the 3-month period prior to baseline and the whole 52-week trial period; vii) cost-effectiveness combining quality of life and health service use and medication use.

Other measures

Prior belief about the effectiveness of the intervention and online resource use (websites or apps) for eczema will be measured at baseline and will be used in a planned subgroup analysis to explore

For peer review only - http://bmjopen.bmj.com/site/about/guidelines.xhtml

whether there is an interaction between prior belief, online resource use and treatment effectiveness.

Process measures

Self-reported barriers to adherence to eczema treatments will be measured at 24-weeks and 52-weeks using the Problematic Experiences of Therapy Scale (PETS)[34] and frequency of eczema treatment use (treatment adherence) will be measured by self-report. Intervention usage data for each participant will be automatically recorded by LifeGuide Software for the duration of the 52-week trial period.

Internal pilot phase

The first 3-months of participant recruitment was an internal pilot phase to test trial procedures, which mirrored the main trial protocol exactly. We assessed study uptake, recruitment and follow-up procedures, randomisation, and participant engagement in accessing the intervention. Success criteria for the pilot phase are listed in the full protocol (available from ECO website [14]).

Data collection methods and retention

All study procedures are automated and carried out online through the LifeGuide software[16]. Participants wishing to take part in the study provide consent and assent (where required) and complete an online baseline questionnaire before being randomised to either the usual care group or the intervention group. Participants in the intervention group then have access to the intervention website (either ECO-YP or ECO-PC).

All participants are asked to complete a 4-weekly POEM questionnaire online for 52 weeks. Participants are also asked to complete a longer 24-week and 52-week follow-up questionnaire online. When signing up for the trial participants are asked if they would prefer reminders by email, text message, or both. Automated emails and/or text messages are sent to notify participants when their follow-up questionnaires are available for completion. Reminders will be sent to non-responders after 5 days (and after 10 days for 24 and 52 week questionnaires), followed by reminder telephone calls approximately 4 days later from the research team, at which point participants will be invited to complete selected follow-up questions over the phone.

Sample size

The sample size calculation for ECO-YP and ECO-PC is based on 4-weekly POEM scores using repeated measures over the first 24 weeks of the trial, seeking to detect a minimum clinically important difference (MCID) of 2.5 points between groups (s.d. 6.5). Assuming a correlation between repeated measures of 0.70, with 90% power and 5% significance, this requires a total sample size of 121 per group in each of the two trials. Allowing for 20% loss to follow up gives a total sample size of 303 in each of the two trials. The sample size was amended during the trial, see protocol amendments section for details.

Statistical analysis plan

Primary analyses of trials ECO-YP and ECO-PC will be generalised linear mixed models, allowing for observations nested within participants over time. All analyses will control for key covariates, including age and baseline eczema severity, and will be set out in full in the Statistical Analysis Plan prior to database lock. For secondary outcome measures, linear models will be used for continuous outcomes. Where the assumptions for linear models are not met, we will use other appropriate distributions or non-parametric methods if no suitable distribution can be found. Logistic regression will be used for binary outcome measures.

For peer review only - http://bmjopen.bmj.com/site/about/guidelines.xhtml

We will collect data on use of other websites at the start and end of the trials to check whether there is a difference between groups in accessing other eczema sites and plan sensitivity analyses to examine whether accessing other resources affects outcomes. All trials of online interventions must assume that users in both groups may access other websites, and so trials provide a useful test of whether the intervention being evaluated is superior to the websites users can already access.

All analyses will be on an intention to treat basis (analysed as randomised), detailed in a Statistical Analysis Plan, and include participants from the internal pilots and full RCTs. No interim analyses are planned. The structure and pattern of missing data will be examined, if appropriate, and a sensitivity analysis based on data imputed using a multiple imputation model presented. Findings will be reported in accordance with the CONSORT statement.

Health economic evaluation

Two within trial economic evaluations will estimate whether ECO-YP and ECO-PC are cost-effective compared to usual care from an NHS and personal social services perspective. We will estimate the cost of the interventions and collect data on wider resource use (primary care, secondary care and accident and emergency use) and eczema-related prescriptions through medical notes review. Resource items will be valued using published unit costs for the most recent common price year to the time of analysis.

There is currently no agreed approach to valuing health outcomes in children in economic evaluations and there has been limited use of child and adolescent population-specific measures to generate health state utilities in NICE technology assessments.[35] In ECO-PC for parents/carers of children aged 0-12 we will collect by proxy the CHU-9D[36], a paediatric generic preference based instrument, in those aged 2 and over. Although the CHU-9D was developed for children aged 7 and over, its completion by proxy in younger age groups is currently being trialled [36] and the developer of the instrument has given us additional guidance to use with parents/carers with children in this age group. This approach is being taken as only parents/carers are expected to interact with the intervention.

In the ECO-YP (young people aged 13-25), all participants are asked to self-complete the EQ-5D-5L in order to estimate their health-related quality of life. To prevent any discontinuity, the EQ-5D-Y will not be used in those under the age of 16 as this is a different instrument to the EQ-5D-5L [37,38]. All participants will be asked to complete the EQ-5D-5L at baseline, 24 weeks and 52 weeks and the scores from these will be converted to utility scores using UK preference weights in line with current recommendations at the time of the analysis [37,39]. Following this, the utility values will be used to estimate Quality-Adjusted Life years (QALY) for the trial period using linear interpolation and area under the curve with and without baseline adjustment.[40]

Cost effectiveness (using change in POEM between baseline and 52 weeks, secondary analysis) and cost utility analyses (primary analysis) will be performed. Costs and benefits will not be discounted given the 12-month timeframe. Using information on costs and benefits, regression analysis will be conducted to estimate the incremental cost, incremental benefit and incremental cost utility of the online intervention compared to usual care (over the trial period). If one arm is clearly dominant (less costly and more effective) a recommendation can be made on this basis. If non-dominance occurs (that is if costs are greater and the intervention is more effective or if the intervention is cheaper and less effective), an incremental cost-effectiveness ratio (ICER) will be produced and a judgement about value for money will need to be made. The economic evaluation will be undertaken and analysed in line with guidelines.[41,42] Missing data will be dealt with in line with the approach taken in the main clinical statistical analysis, with sensitivity analysis undertaken to test the impact of approach if missing data is a particular problem. A detailed Health Economic Analysis Plan will be written and reviewed before the trial database is locked.

For peer review only - http://bmjopen.bmj.com/site/about/guidelines.xhtml

Nested process evaluation

The nested process evaluation studies are being carried out to understand intervention processes and participants' experiences of using the interventions.

Quantitative process evaluation

We will use baseline data to examine potential predictors and moderator effects of participant characteristics (e.g. age, eczema symptom severity, baseline attitudes) on intervention engagement (objectively recorded detailed website usage and self-reported treatment adherence) and outcome. We will also assess and analyse hypothesised mediators of treatment adherence and intervention outcomes; specifically changes in beliefs about treatment (PETS) as well as intervention usage. Objective measures of intervention usage are automatically recorded (with informed participant consent), allowing evaluations of usage patterns, such as time spent on intervention, number of visits to the intervention website, and pages visited.

Qualitative process evaluation

Qualitative process interviews will be carried out with approximately 30-40 participants (15 to 20 from ECO-YP and 15-20 from ECO-PC, or until saturation of the main themes are achieved). These interviews will provide in-depth understanding of patient and carers' experiences within the trial and provide a better understanding of factors that may influence engagement.

Interviews will be conducted via telephone or video call by a member of the research team experienced in qualitative research methods. We will interview participants from the intervention group and the usual care group and use purposive sampling to ensure a range of age, gender, ethnicity, eczema severity, website usage, deprivation index, and region. Potential participants will be contacted after being in the trial for at least 3 months by a member of the research team to check whether they would like to take part in an interview or have any questions about the study. Participants will be asked to give their consent online prior to the interview. Interviews will use a combination of open-ended and focussed questions and be transcribed verbatim.

Qualitative data will first be analysed using inductive thematic analysis.[43] We will then explore how emerging themes may map onto theoretical frameworks in order to relate our insights to generalisable theoretical constructs and inform implementation planning.

Process evaluation analysis

We will triangulate findings from the quantitative and qualitative process analyses [44] to explore and test the causal mechanisms proposed, to help inform interpretation of trial results, and determine how the interventions could be improved and how implementation into clinical practice could be facilitated.

Patient and Public Involvement (PPI)

The study team includes two PPI members (AR and AA) who have been involved in the project from the earliest stages. They are involved in all aspect of the ECO programme and trials, including intervention development[22], trial design, attending trial and programme management meetings, protocol discussions, developing participant facing materials, and co-authoring outputs. Our PPI partners will also be key for dissemination and future implementation.

ETHICS AND DISSEMINATION

For peer review only - http://bmjopen.bmj.com/site/about/guidelines.xhtml

The study has received the favourable opinion of South Central – Oxford A Research Ethics Committee (19/SC/0351). This summary protocol is based on approved protocol v3 (20/05/2020), ISRCTN reference 79282252.

Data monitoring

An independent Programme Steering Committee take responsibility for safeguarding the interest of study participants, monitor the main outcome measures including safety and efficacy, and monitor the overall conduct of the trials.

Protocol amendments

One substantial protocol amendment has been made since initial ethics approval (Substantial Amendment 1, number 47369.A4, approved 01/06/20). This amendment was in response to the Covid19 pandemic in order to:

1. Make all trial process online. The original protocol required parents or guardians of 13-15-year olds to return parental consent by post.
2. Increase in sample size. Our original sample size was 200 participants per trial, based on the published POEM MCID of 3[45]. However, research has since suggested that a smaller POEM MCID may be meaningful in certain contexts [46]. Recruitment to both trials exceeded expectation and a protocol amendment was made to change the sample size to a minimum of '200 participants' per trial to allow us to continue recruitment while a revised sample size was discussed with our Trial Management Group, Programme Management Group, Programme Steering Committee, and funder, without access to study outcome data or any interim analysis. The final agreed sample size for the trials were based on seeking to detect a POEM MCID of 2.5 points between groups, based on two repeated measures (s.d. 6.5), allowing for 20% loss to follow-up requiring sample size of 303 participants in each of the two trials.

Dissemination

As a minimum, study progress, outputs and trial findings will be made available via the study website[14] and project twitter (@ECO_eczema). Summaries will also be sent to participants and participating GP surgeries. Findings will be presented at conferences and published in peer-reviewed journals. We will make available a deidentified data set on request.

For peer review only - http://bmjopen.bmj.com/site/about/guidelines.xhtml

AUTHOR CONTRIBUTIONS

MS and KT conceived the study idea and initial study design in collaboration with IM, LY, PLi, HW, JC, MR, SLa, BS, GG, TS, SiL, AR, AA, HK, with later input from JN, JH, SW, MSt, KG, KS, TB. Specific advice was given by BS and TB on trial design and medical statistics; IM, LY, KG, KS, PLe, LH on the process evaluation; and TS on the health economic evaluation. All the authors contributed to the drafting of the study protocol, led by IM, and approved the final manuscript.

FUNDING STATEMENT

This study presents independent research funded by the National Institute for Health Research (NIHR) under its Programme Grants for Applied Research programme (grant ref No RP-PG-0216-20007). Eczema Care Online (ECO) interventions were developed using LifeGuide software, which was partly funded by the NIHR Southampton Biomedical Research Centre (BRC). SiL was supported by a Wellcome senior research fellowship in clinical science (205039/Z/16/Z).

The views expressed are those of the author(s) and not necessarily those of the NHS, the NIHR or the Department of Health and Social Care.

COMPETING INTERESTS

No competing interests to declare

For peer review only

For peer review only - http://bmjopen.bmj.com/site/about/guidelines.xhtml

REFERENCES

1. Chamlin SL, Frieden IJ, Williams ML, et al. Effects of atopic dermatitis on young American children and their families. *Pediatrics* 2004;114(3):607-11.
2. Noerreslet M, Jemec GB, Traulsen JM. Involuntary autonomy: patients' perceptions of physicians, conventional medicines and risks in the management of atopic dermatitis. *Social Science & Medicine.69(9):1409-15* 2009.
3. Mollerup A, Johansen JD, Thing LF. Knowledge, attitudes and behaviour in everyday life with chronic hand eczema: a qualitative study. *Br J Dermatol* 2013;169(5):1056-65.
4. NICE. Clinical guideline 57. Atopic eczema in under 12s: diagnosis and management, 2007.
5. Bass AM, Anderson KL, Feldman SR. Interventions to Increase Treatment Adherence in Pediatric Atopic Dermatitis: A Systematic Review. *J Clin Med* 2015;4(2):231-42.
6. Beattie PE, Lewis-Jones MS. Parental knowledge of topical therapies in the treatment of childhood atopic dermatitis. *Clin Exp Dermatol* 2003;28:549-53.
7. Santer M, Burgess H, Yardley L, et al. Managing childhood eczema: qualitative study exploring carers' experiences of barriers and facilitators to treatment adherence. *J Adv Nurs* 2013;69(11):8.
8. Charman CR, Morris AD, Williams HC. Topical corticosteroid phobia in patients with atopic eczema. *British Journal of Dermatology* 2000;142(5):931-36.
9. Santer M, Burgess H, Yardley L, et al. Experiences of carers managing childhood eczema and their views on its treatment: a qualitative study. *British Journal of General Practice* 2012;62:261-67.
10. Powell K, Le Roux E, Banks J, et al. GP and parent dissonance about the assessment and treatment of childhood eczema in primary care: a qualitative study. *BMJ Open* 2018;8(2).
11. Horne R, Chapman SC, Parham R, et al. Understanding patients' adherence-related beliefs about medicines prescribed for long-term conditions: a meta-analytic review of the Necessity-Concerns Framework. *PLoS ONE [Electronic Resource]* 2013;8(12):e80633.
12. Santer M, Muller I, Yardley L, et al. 'You don't know which bits to believe': qualitative study exploring carers' experiences of seeking information on the internet about childhood eczema. *BMJ Open* 2015;5(4):e006339.
13. OfN S. Internet access – households and individuals, Great Britain: 2020. 2020.
14. ECO. *Eczema Care Online project website*. www.nottingham.ac.uk/eco (accessed 21/09/20).
15. Van den Haak MJ, De Jong MD, Schellens PJ. Evaluation of an informational web site: three variants of the think-aloud method compared. *Technical Communication* 2007;54(1):58-71.
16. Williams S, Yardley L, Weal M, et al. Introduction to LifeGuide: Open-source software for creating online interventions for health care, health promotion and training. *Knowledge Resources* 2010;187:90.
17. Yardley L, Morrison L, Bradbury K, et al. The person-based approach to intervention development: application to digital health-related behavior change interventions. *Journal of Medical Internet Research* 2015;17(1).
18. Morrison L, Muller I, Yardley L, et al. The person-based approach to planning, optimising, evaluating and implementing behavioural health interventions. *The European Health Psychologist* 2018;20(3):464-69.
19. Hagger MS, Orbell S. A meta-analytic review of the common-sense model of illness representations. *Psychology and health* 2003;18(2):141-84.
20. Bandura A. Self-efficacy: Towards a unifying theory and the organization. *Psychological Review* 1977;84:191-215.
21. Michie S, Van Stralen MM, West R. The behaviour change wheel: a new method for characterising and designing behaviour change interventions. *Implementation Science* 2011;6(1):42.

For peer review only - http://bmjopen.bmj.com/site/about/guidelines.xhtml

22. Muller I, Santer M, Morrison L, et al. Combining qualitative research with PPI: reflections on using the person-based approach for developing behavioural interventions. *Research Involvement and Engagement* 2019;5(1):34.
23. Hoffmann TC, Glasziou PP, Boutron I, et al. Better reporting of interventions: template for intervention description and replication (TIDieR) checklist and guide. *BMJ* 2014;348.
24. Spuls PI, Gerbens L, Simpson E, et al. Patient-Oriented Eczema Measure (POEM), a core instrument to measure symptoms in clinical trials: a Harmonising Outcome Measures for Eczema (HOME) statement. *British Journal of Dermatology* 2017;176(4):979-84.
25. CEBD. *Harmonising Outcome Measures for Eczema (HOME)*. http://www.homeforeczema.org (accessed 21/09/20).
26. Charman C, Venn AJ, Williams HC. The patient-oriented eczema measure: development and initial validation of a new tool for measuring atopic eczema severity from the patients' perspective. *Archives of dermatology* 2004;140 (12):1513-19.
27. Charman C, Venn AJ, Ravenscroft JC, et al. Translating Patient-Oriented Eczema Measure (POEM) scores into clinical practice by suggesting severity strata derived using anchor-based methods, 2013:1326-32.
28. Schmitt J, Langan S, Deckert S, et al. Assessment of clinical signs of atopic dermatitis: A systematic review and recommendation. *Journal of Allergy and Clinical Immunology* 2013;132(6):1337-47.
29. Herdman M, Gudex C, Lloyd A, et al. Development and preliminary testing of the new five-level version of EQ-5D (EQ-5D-5L). *Qual Life Res* 2011;20(10):1727-36.
30. Stevens K. Valuation of the child health utility 9D index. *Pharmacoeconomics* 2012;30(8):729-47.
31. Howells L, Chalmers J, Gran S, et al. Development and initial testing of a new instrument to measure the experience of eczema control in adults and children: Recap of atopic eczema (RECAP). *British Journal of Dermatology* 2020;183(3):524-36.
32. Yosipovitch G, Reaney M, Mastey V, et al. Peak Pruritus Numerical Rating Scale: psychometric validation and responder definition for assessing itch in moderate-to-severe atopic dermatitis. *British Journal of Dermatology* 2019;181(4):761-69.
33. Howie JGR, Heaney DJ, M. M, et al. A Comparison of a Patinet Enablement Intrument (PEI) Against Two Established Satisfaciton Scales as an Outcome Measure of Primary Care Consultation. *Family Practice* 1998;15(2):165-71.
34. Kirby S, Donovan-Hall M, Yardley L. Measuring barriers to adherence: validation of the Problematic Experiences of Therapy Scale. *Disabil Rehabil* 2014;36(22):1924-9.
35. Ungar W. *Economic evaluation in child health*: Oxford University Press; 2009.
36. Canaway AG, Frew EJ. Measuring preference-based quality of life in children aged 6-7 years: a comparison of the performance of the CHU-9D and EQ-5D-Y--the WAVES pilot study. *Qual Life Res* 2013;22(1):173-83.
37. Devlin N SK, Feng Y, Mulhern B. van Hout, B. *Valuing Health-Related Quality of Life: An EQ-5D-5L Value Set for England*. https://www.sheffield.ac.uk/scharr/sections/heds/discussion-papers/16-02-1.546901 (accessed 6 April).
38. EuroQOL. *EQ-5D-Y User Guide V1.0*. https://euroqol.org/wp-content/uploads/2016/09/EQ-5D-Y_User_Guide_v1.0_2014.pdf (accessed 13 December).
39. NICE. *Position statement on use of the EQ-5D-5L valuation set.* . https://www.nice.org.uk/Media/Default/About/what-we-do/NICE-guidance/NICE-technology-appraisal-guidance/eq5d5l_nice_position_statement.pdf (accessed 20 August).
40. Manca A, Hawkins N, Sculpher MJ. Estimating mean QALYs in trial-based cost-effectiveness analysis: the importance of controlling for baseline utility. *Health Econ* 2005;14(5):487-96.
41. Ramsey SD, Willke RJ, Glick H, et al. Cost-Effectiveness Analysis Alongside Clinical Trials II—An ISPOR Good Research Practices Task Force Report. *Value in Health* 2015;18(2):161-72.
42. Drummond MF, Sculpher M, Claxton K, et al. *Methods for the economic evaluation of health care programmes*. 4th edition ed. Oxford: Oxford University Press; 2015.

For peer review only - http://bmjopen.bmj.com/site/about/guidelines.xhtml

43. Braun V, Clarke V. Using thematic analysis in psychology. *Qualitative Research in Psychology* 2006;3(2):77-101.
44. Yardley L, Bishop F. Mixing qualitative and quantitative methods: A pragmatic approach. In: Willig C, Stainton-Rogers W (eds.) *Qualitative Research in Psychology*. Los Angeles, USA: Sage; 2007 p313-26.
45. Schram ME, Spuls PI, Leeflang MM, et al. EASI, (objective) SCORAD and POEM for atopic eczema: responsiveness and minimal clinically important difference. *Allergy* 2012;67(1):99-106.
46. Howells L, Ratib S, Chalmers J, et al. How should minimally important change scores for the Patient-Oriented Eczema Measure be interpreted? A validation using varied methods. *British Journal of Dermatology* 2018;178(5):1135-42.

For peer review only

Table 1. Schedule of observations.

Outcomes collected	Baseline	24 weeks (primary outcome)	52 Weeks (end of study)
**Baseline characteristics**			
Demographics	✓		
Prior belief about effectiveness	✓		
Previous online resource use	✓		
**Clinical effectiveness outcomes**			
POEM (4-weekly)	✓	✓	✓
Long-term control (Recap)	✓	✓	✓
Itch intensity measure (ECO-YP only)	✓	✓	✓
Patient enablement Instrument (PEI)	✓	✓	✓
**Cost effectiveness outcomes**			
CHU-9D (ECO-PC for parents / carers of children aged 2-12 only)	✓	✓	✓
EQ-5D-5L (ECO-YP only)	✓	✓	✓
Medical notes review for medication use, service use, and referrals			✓ (including 3 months pre-baseline period)
**Process outcomes**			
Problematic Experiences of Therapy Scale (PETS)	✓	✓	✓
Frequency of eczema treatment use (adherence)	✓	✓	✓
Intervention usage			✓ (recorded throughout study)

For peer review only - http://bmjopen.bmj.com/site/about/guidelines.xhtml

Figure legends

Figure 1. Flow of participants through the trial

For peer review only

For peer review only - http://bmjopen.bmj.com/site/about/guidelines.xhtml

Figure 1. Flow of participants through the trial

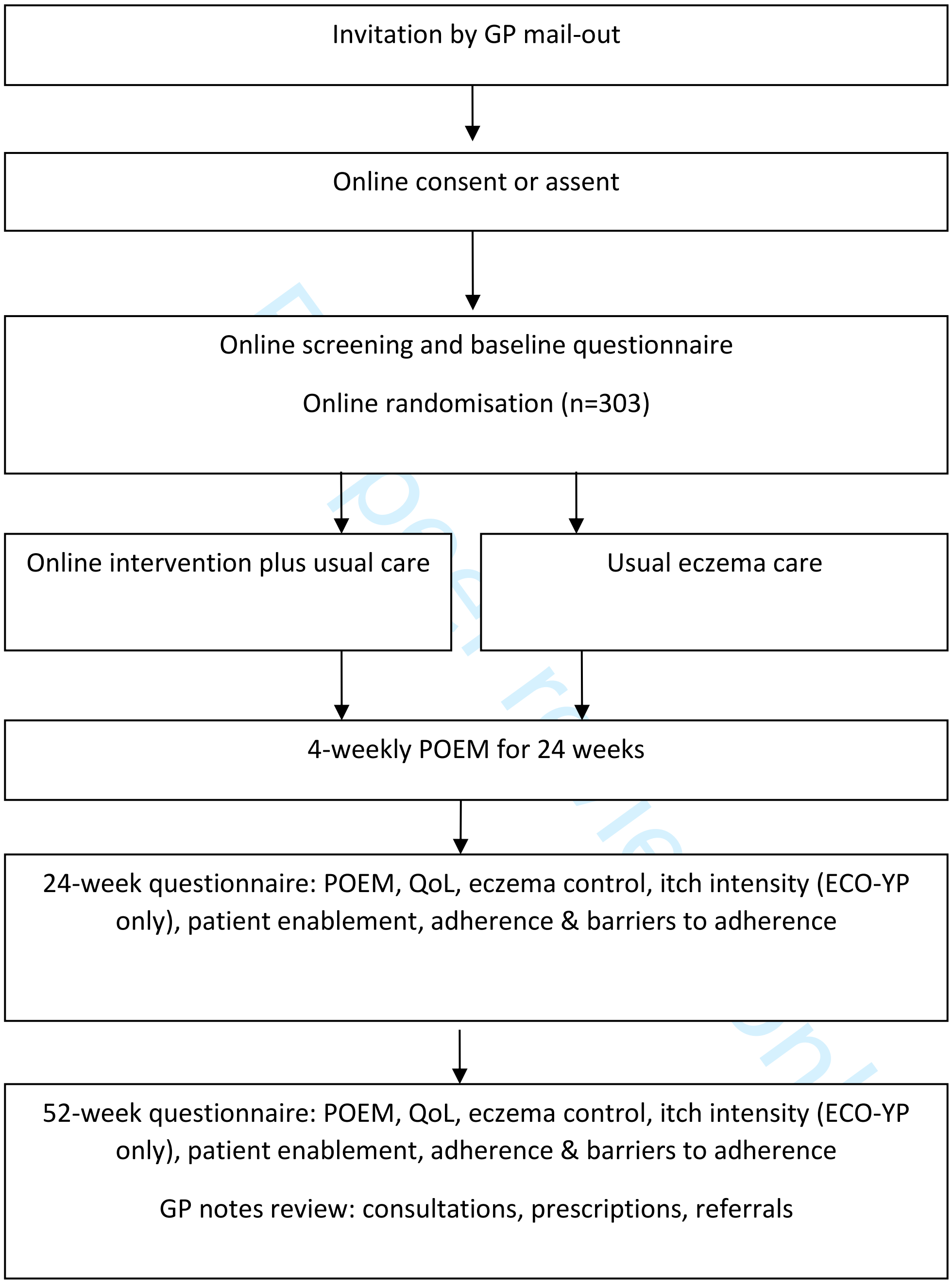

For peer review only - http://bmjopen.bmj.com/site/about/guidelines.xhtml

STANDARD PROTOCOL ITEMS: RECOMMENDATIONS FOR INTERVENTIONAL TRIALS

SPIRIT 2013 Checklist: Recommended items to address in a clinical trial protocol and related documents*

Section/item	Item No	Description	Addressed on page number
**Administrative information**			
Title	1	Descriptive title identifying the study design, population, interventions, and, if applicable, trial acronym	___1_________
Trial registration	2a	Trial identifier and registry name. If not yet registered, name of intended registry	___2_________
	2b	All items from the World Health Organization Trial Registration Data Set	See protocol on website
Protocol version	3	Date and version identifier	See protocol on website
Funding	4	Sources and types of financial, material, and other support	_____13_______
Roles and responsibilities	5a	Names, affiliations, and roles of protocol contributors	_____1 & 13_______
	5b	Name and contact information for the trial sponsor	See protocol on website
	5c	Role of study sponsor and funders, if any, in study design; collection, management, analysis, and interpretation of data; writing of the report; and the decision to submit the report for publication, including whether they will have ultimate authority over any of these activities	See protocol on website
	5d	Composition, roles, and responsibilities of the coordinating centre, steering committee, endpoint adjudication committee, data management team, and other individuals or groups overseeing the trial, if applicable (see Item 21a for data monitoring committee)	_____10_______

For peer review only

Introduction

Background and rationale	6a	Description of research question and justification for undertaking the trial, including summary of relevant studies (published and unpublished) examining benefits and harms for each intervention	__3__________
	6b	Explanation for choice of comparators	__3__________
Objectives	7	Specific objectives or hypotheses	__3__________
Trial design	8	Description of trial design including type of trial (eg, parallel group, crossover, factorial, single group), allocation ratio, and framework (eg, superiority, equivalence, noninferiority, exploratory)	__3__________

Methods: Participants, interventions, and outcomes

Study setting	9	Description of study settings (eg, community clinic, academic hospital) and list of countries where data will be collected. Reference to where list of study sites can be obtained	__4__________
Eligibility criteria	10	Inclusion and exclusion criteria for participants. If applicable, eligibility criteria for study centres and individuals who will perform the interventions (eg, surgeons, psychotherapists)	__4__________
Interventions	11a	Interventions for each group with sufficient detail to allow replication, including how and when they will be administered	__5__________
	11b	Criteria for discontinuing or modifying allocated interventions for a given trial participant (eg, drug dose change in response to harms, participant request, or improving/worsening disease)	__n/a__________
	11c	Strategies to improve adherence to intervention protocols, and any procedures for monitoring adherence (eg, drug tablet return, laboratory tests)	__6__________
	11d	Relevant concomitant care and interventions that are permitted or prohibited during the trial	__5__________
Outcomes	12	Primary, secondary, and other outcomes, including the specific measurement variable (eg, systolic blood pressure), analysis metric (eg, change from baseline, final value, time to event), method of aggregation (eg, median, proportion), and time point for each outcome. Explanation of the clinical relevance of chosen efficacy and harm outcomes is strongly recommended	__6__________
Participant timeline	13	Time schedule of enrolment, interventions (including any run-ins and washouts), assessments, and visits for participants. A schematic diagram is highly recommended (see Figure)	Figure 1, p11

For peer review only - http://bmjopen.bmj.com/site/about/guidelines.xhtml

Sample size	14	Estimated number of participants needed to achieve study objectives and how it was determined, including clinical and statistical assumptions supporting any sample size calculations	___8__________
Recruitment	15	Strategies for achieving adequate participant enrolment to reach target sample size	___7_________

Methods: Assignment of interventions (for controlled trials)

Allocation:

Sequence generation	16a	Method of generating the allocation sequence (eg, computer-generated random numbers), and list of any factors for stratification. To reduce predictability of a random sequence, details of any planned restriction (eg, blocking) should be provided in a separate document that is unavailable to those who enrol participants or assign interventions	___5_________
Allocation concealment mechanism	16b	Mechanism of implementing the allocation sequence (eg, central telephone; sequentially numbered, opaque, sealed envelopes), describing any steps to conceal the sequence until interventions are assigned	___5_________
Implementation	16c	Who will generate the allocation sequence, who will enrol participants, and who will assign participants to interventions	___5_________
Blinding (masking)	17a	Who will be blinded after assignment to interventions (eg, trial participants, care providers, outcome assessors, data analysts), and how	___5______
	17b	If blinded, circumstances under which unblinding is permissible, and procedure for revealing a participant's allocated intervention during the trial	Not applicable

Methods: Data collection, management, and analysis

Data collection methods	18a	Plans for assessment and collection of outcome, baseline, and other trial data, including any related processes to promote data quality (eg, duplicate measurements, training of assessors) and a description of study instruments (eg, questionnaires, laboratory tests) along with their reliability and validity, if known. Reference to where data collection forms can be found, if not in the protocol	____7_________
	18b	Plans to promote participant retention and complete follow-up, including list of any outcome data to be collected for participants who discontinue or deviate from intervention protocols	____7________

Data management	19	Plans for data entry, coding, security, and storage, including any related processes to promote data quality (eg, double data entry; range checks for data values). Reference to where details of data management procedures can be found, if not in the protocol	____7________
Statistical methods	20a	Statistical methods for analysing primary and secondary outcomes. Reference to where other details of the statistical analysis plan can be found, if not in the protocol	____8________
	20b	Methods for any additional analyses (eg, subgroup and adjusted analyses)	____8________
	20c	Definition of analysis population relating to protocol non-adherence (eg, as randomised analysis), and any statistical methods to handle missing data (eg, multiple imputation)	____8________
**Methods: Monitoring**			
Data monitoring	21a	Composition of data monitoring committee (DMC); summary of its role and reporting structure; statement of whether it is independent from the sponsor and competing interests; and reference to where further details about its charter can be found, if not in the protocol. Alternatively, an explanation of why a DMC is not needed	____10_______
	21b	Description of any interim analyses and stopping guidelines, including who will have access to these interim results and make the final decision to terminate the trial	___7_________
Harms	22	Plans for collecting, assessing, reporting, and managing solicited and spontaneously reported adverse events and other unintended effects of trial interventions or trial conduct	See protocol on website
Auditing	23	Frequency and procedures for auditing trial conduct, if any, and whether the process will be independent from investigators and the sponsor	See protocol on website
**Ethics and dissemination**			
Research ethics approval	24	Plans for seeking research ethics committee/institutional review board (REC/IRB) approval	____9________
Protocol amendments	25	Plans for communicating important protocol modifications (eg, changes to eligibility criteria, outcomes, analyses) to relevant parties (eg, investigators, REC/IRBs, trial participants, trial registries, journals, regulators)	____10_______

Consent or assent	26a	Who will obtain informed consent or assent from potential trial participants or authorised surrogates, and how (see Item 32)	_____4________
	26b	Additional consent provisions for collection and use of participant data and biological specimens in ancillary studies, if applicable	Not applicable
Confidentiality	27	How personal information about potential and enrolled participants will be collected, shared, and maintained in order to protect confidentiality before, during, and after the trial	See protocol on website
Declaration of interests	28	Financial and other competing interests for principal investigators for the overall trial and each study site	____13__________
Access to data	29	Statement of who will have access to the final trial dataset, and disclosure of contractual agreements that limit such access for investigators	________________
Ancillary and post-trial care	30	Provisions, if any, for ancillary and post-trial care, and for compensation to those who suffer harm from trial participation	________________
Dissemination policy	31a	Plans for investigators and sponsor to communicate trial results to participants, healthcare professionals, the public, and other relevant groups (eg, via publication, reporting in results databases, or other data sharing arrangements), including any publication restrictions	_____10_________
	31b	Authorship eligibility guidelines and any intended use of professional writers	See protocol on website
	31c	Plans, if any, for granting public access to the full protocol, participant-level dataset, and statistical code	Full protocol is on study website.
**Appendices**			
Informed consent materials	32	Model consent form and other related documentation given to participants and authorised surrogates	________________
Biological specimens	33	Plans for collection, laboratory evaluation, and storage of biological specimens for genetic or molecular analysis in the current trial and for future use in ancillary studies, if applicable	________________

*It is strongly recommended that this checklist be read in conjunction with the SPIRIT 2013 Explanation & Elaboration for important clarification on the items. Amendments to the protocol should be tracked and dated. The SPIRIT checklist is copyrighted by the SPIRIT Group under the Creative Commons "Attribution-NonCommercial-NoDerivs 3.0 Unported" license.

For peer review only - http://bmjopen.bmj.com/site/about/guidelines.xhtml
