# Supplementary material for: Supporting self-care for eczema: protocol for two randomised controlled trials of ECO (Eczema Care Online) interventions for young people and parents/carers
Source: BMJ Open. 2021 Feb 5;11(2):e045583. doi: 10.1136/bmjopen-2020-045583 (PMC7925854; doi:10.1136/bmjopen-2020-045583)
Supplement: Reviewer comments [file bmjopen-2020-045583.reviewer_comments.pdf]

## PEER REVIEW HISTORY

BMJ Open publishes all reviews undertaken for accepted manuscripts. Reviewers are asked to complete a checklist review form (**http://bmjopen.bmj.com/site/about/resources/checklist.pdf**) and are provided with free text boxes to elaborate on their assessment. These free text comments are reproduced below.

## ARTICLE DETAILS

| | |
|---|---|
| **TITLE (PROVISIONAL)** | Supporting self-care for eczema: protocol for two randomised controlled trials of ECO (Eczema Care Online) interventions for young people and parents/carers |
| **AUTHORS** | Muller, Ingrid; Stuart, Beth; Sach, Tracey; Hooper, Julie; Wilczynska, Sylvia; Steele, Mary; Greenwell, Kate; Sivyer, Katy; Yardley, Lucy; Williams, HC; Chalmers, Joanne; Leighton, Paul; Howells, Laura; Ridd, Matthew; Lawton, Sandra; Griffiths, Gareth; Nuttall, Jacqui; Langan, Sinead; Roberts, Amanda; Ahmed, Amina; Kirk, Hayden; Becque, Taeko; Little, Paul; Thomas, Kim; Santer, Miriam |

This article was not externally reviewed at BMJ Open. Protocols that have been independently assessed prior to submission to BMJ Open are usually fast-tracked to publication on the grounds that further substantial changes will not be possible. This independent assessment will usually be external, independent review for both a substantial grant award from a non-commercial or government funder and ethics approval.
